# New ancient Eastern European *Yersinia pestis* genomes illuminate the dispersal of plague in Europe

**DOI:** 10.1098/rstb.2019.0569

**Published:** 2020-10-05

**Authors:** Irina Morozova, Artem Kasianov, Sergey Bruskin, Judith Neukamm, Martyna Molak, Elena Batieva, Aleksandra Pudło, Frank J. Rühli, Verena J. Schuenemann

**Affiliations:** 1 Institute of Evolutionary Medicine, University of Zurich, Winterthurerstrasse 190, 8057 Zurich, Switzerland; 2 Vavilov Institute of General Genetics, Russian Academy of Sciences, Gubkina Street 3, Moscow 119991, Russia; 3 Laboratory of Plant Genomics, The Institute for Information Transmission Problems RAS, Moscow 127051, Russia; 4 Institute for Bioinformatics and Medical Informatics, University of Tübingen, Sand 14, 72076 Tübingen, Germany; 5 Museum and Institute of Zoology, Polish Academy of Sciences, Wilcza 64, Warsaw 00-679, Poland; 6 Centre of New Technologies, University of Warsaw, S. Banacha 2c, Warsaw 02-097, Poland; 7 Azov History, Archeology and Paleontology Museum-Reserve, Moskovskaya Street 38/40, Azov 346780, Russia; 8 Archaeological Museum in Gdańsk, Mariacka Street 25/26, Gdańsk 80-833, Poland

**Keywords:** plague, *Yersinia pestis*, pathogen evolution, ancient pathogen genomics, ancient DNA

## Abstract

*Yersinia pestis*, the causative agent of plague, has been prevalent among humans for at least 5000 years, being accountable for several devastating epidemics in history, including the Black Death. Analyses of the genetic diversity of ancient strains of *Y. pestis* have shed light on the mechanisms of evolution and the spread of plague in Europe. However, many questions regarding the origins of the pathogen and its long persistence in Europe are still unresolved, especially during the late medieval time period. To address this, we present four newly assembled *Y. pestis* genomes from Eastern Europe (Poland and Southern Russia), dating from the fifteenth to eighteenth century AD. The analysis of polymorphisms in these genomes and their phylogenetic relationships with other ancient and modern *Y. pestis* strains may suggest several independent introductions of plague into Eastern Europe or its persistence in different reservoirs. Furthermore, with the reconstruction of a partial *Y. pestis* genome from rat skeletal remains found in a Polish ossuary, we were able to identify a potential animal reservoir in late medieval Europe. Overall, our results add new information concerning *Y. pestis* transmission and its evolutionary history in Eastern Europe.

This article is part of the theme issue ‘Insights into health and disease from ancient biomolecules’.

## 1. Introduction

*Yersinia pestis*, the causative agent of plague, is well known as an infectious agent responsible for the most devastating epidemics in Europe [1]. In the past, three major plague pandemics killed up to 60% of the population in the Old World [2,3]. The first pandemic, known as the Plague of Justinian, began in 541–544 AD and continued intermittently until *ca* 750 AD [1–3]. It affected the Eastern Roman Empire, the Sasanian Empire and port cities around the Mediterranean Sea [1–3]. The second pandemic in Europe began with the Black Death (1347–1351 AD) and continued with several successive waves until the eighteenth century [1–3]. The third pandemic originated in China in 1855 and erupted there into a major epidemic, then spread all over the world and incited a series of epidemics until the middle of the twentieth century [1–3]. Often considered as a historical relic, plague is still a viable threat with worldwide outbreaks [4–6]. Since 2000, more than twenty outbreaks have been documented across the world; the last severe one in 2017 occurred in Madagascar [4,6]. Natural reservoirs of plague infection are present in Central, Eastern and Southern Africa, South America, the western part of North America and in large areas of Asia. These reservoirs are considered the main reason for the impossibility of plague eradication [5], and include ground squirrels, rabbits, hares and other animals [2,3]. Additionally, peri-domestic animals are often a source of infection for humans via fleas living on infected rats or via direct contact with wild or other peri-domestic animals [2,3].

Recent studies have demonstrated that the evolution of *Y. pestis* was very complex and likely triggered by not only host–pathogen interactions but also massive human migrations [2,7–9]. The gain and loss of various genes and associated virulent features of *Y. pestis* likely took place more than once [9]. The availability of many ancient and modern *Y. pestis* genomes has facilitated the reconstruction of its global phylogeny [8,10–14].

The avenues in which *Y. pestis* was brought into Europe are still debated, as well as the mechanism of the plague's persistence in Europe for several hundred years [15–17]. According to one theory, *Y. pestis* was repeatedly reintroduced into Europe from Asia with several waves along major trade routes [15]. For this hypothesis to be plausible, high genetic variability reflecting the natural genetic diversity of *Y. pestis* should be detected in different plague victims. The second hypothesis suggests a persistence of *Y. pestis* in Europe for a long time in an unknown reservoir or unidentified host [16,17]. In such a scenario, identical or very similar *Y. pestis* genotypes should be present in plague victims from various different time periods. So far, the analysis of the genetic diversity of *Y. pestis* in plague victims from varying time points during the second pandemic, from the fourteenth to eighteenth century, suggested genetic continuity between *Y. pestis* strains for almost five centuries in Western and Central Europe [18–20]. The researchers proposed that the *Y. pestis* responsible for the Black Death appeared once in some reservoir within Europe, Caucasus or Western Asia, and then evolved locally over several centuries [18,19]; however, thus far, no source reservoir for European plague has been identified. The question about potential animal sources of plague in medieval Europe is still highly debated. Some researchers connect the Black Death with black rats (*Rattus rattus*), while others indicate human fleas or body lice as a more plausible source [21–25].

Eastern Europe is one of the key regions to investigate the spread of plague and the evolutionary mechanisms underpinning it. Being at the interface of Europe and Asia, Eastern Europe is a likely gateway for the introduction of plague into Europe and may contain extremely important information on the circulation and possible ecological niches of *Y. pestis* in the region [26]. However, until now, only two medieval *Y. pestis* genomes were available from Eastern Europe, namely from two burial grounds dated to the thirteenth to fourteenth century from Tatarstan: Bolgar and Laishevo [20,27]. To characterize the genetic diversity of plague in Eastern Europe, we reconstructed four complete *Y. pestis* genomes from skeletal remains belonging to plague victims in Southern Russia (sixteenth to eighteenth century) and Poland (fifteenth to eighteenth century). In addition, we analysed DNA from rat skull fragments from the Gdańsk ossuary (Poland) to obtain genetic data from a potential animal plague reservoir in Europe.

## 2. Material and methods

**Figure 1.**
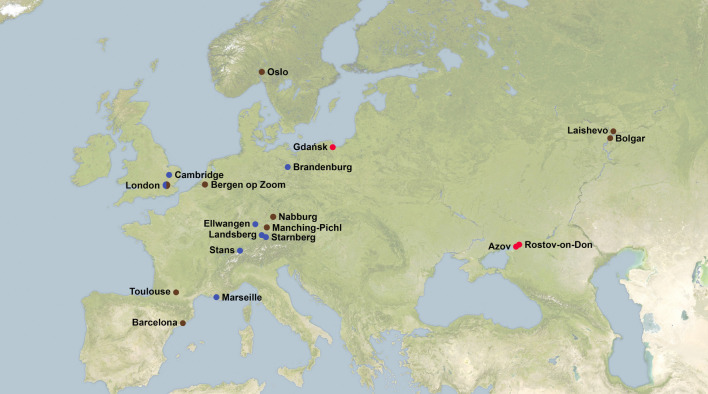
Location of ancient samples from victims of the second and third plague epidemics. Red dots highlight the locations of the samples from our study: Rostov-on-Don, Russia (Rostov2033 and Rostov2039, 1762–1773 AD), Azov (Azov38, fifteenth to seventeenth century AD) and Gdańsk (Gdansk8, 1425–1469 AD). Brown dots represent locations of previously published *Y. pestis* samples closer to the Black Death period (thirteenth to fourteenth century, [12,20,27,28]). Blue dots represent locations of previously published *Y. pestis* samples dated to the post-Black Death period (fifteenth to eighteenth century, [12,18,20,27]). (Online version in colour.)

Human samples (78 in total) were collected from three burial sites in Eastern Europe where plague has been documented (figure 1; electronic supplementary material, table S1): (1) the cemetery from St Dmitry Rostovsky fortress (Rostov-on-Don, Russia) where, according to historical documents, the victims of plague *ca* 1762–1773 were buried (*N* = 39); (2) the fifteenth to seventeenth century burials in Azov city (Rostov-on-Don region, Russia) (*N* = 4); (3) the Gdańsk ossuaries dated to the fifteenth to eighteenth century (*N* = 35) (for details see electronic supplementary material, note S1). In addition, three fragments of one rat skull were collected in one of the Gdańsk ossuaries and combined into one sample for downstream DNA analyses (figure 1; electronic supplementary material, note S1 and table S1).

All samples were screened for the presence of a fragment of the plasminogen activator (*pla*) gene, which is a unique identifier for *Y. pestis* [11] (electronic supplementary material, note S2). Samples positive for the *pla* gene were converted into Illumina double-stranded libraries [29,30] and were shotgun sequenced (electronic supplementary material, note S2). Afterwards, all samples with confirmed *Y. pestis* presence were subjected to targeted enrichment [12,31] using selected *Y. pestis* genomes (NC_003143.1, NC_003131.1, NC_003134.1, NC_003132.1) as a reference for probe design (electronic supplementary material, note S2). The next generation sequencing (NGS) data were used as a source for phylogenetic, genealogical and functional analysis (electronic supplementary material, note S2). For comparative analysis, 257 previously published ancient and modern *Y. pestis* strains ([8,9,12,18,20,27,28,32–37], electronic supplementary material, table S2) were used (electronic supplementary material, note S2).

## 3. Results

### (a) Molecular analysis of the human samples

PCR screening [11,13] of 78 human samples revealed five (four from Southern Russia, one from Poland; electronic supplementary material, table S1) *pla*-positive amplicons. Shotgun sequencing and subsequent MALT (MEGAN Alignment Tool) analysis using all complete bacterial, viral and archaeal genomes in GenBank as a reference [38,39] confirmed the presence of *Y. pestis* DNA in these samples. The damage profiles of *Homo sapiens* DNA (shotgun) and *Y. pestis* (enrichment) showed increased rates of C > T changes (3.4–18.7%) at the terminal ends of DNA fragments [40], thus demonstrating the authenticity of the analysed DNA (electronic supplementary material, figure S1).

Target enrichment [12,31] with probes specific for the *Y. pestis* chromosome and its three plasmids (pCD1, pMT1 and pPCP1), and subsequent high-throughput sequencing, yielded data sufficient for analysis in four out of five *Y. pestis* positive human samples (Rostov2033, Rostov2039, Azov38 and Gdansk8; table 1). The mapping to the *Y. pestis* reference genome (NC 003143.1, NC 003134.1, NC 003131.1, NC 003132.1) revealed 88–96% of genome length coverage for *Y. pestis* chromosome, 67–100% length coverage for the plasmids, and a minimum fourfold to maximum 184-fold mean coverage (9–245-fold for plasmids) (table 1; electronic supplementary material, table S3 and figure S2).

**Table 1.** Next generation sequencing data for plague-positive samples. Results of mapping the untrimmed shotgun and enriched plague-positive samples against different references (*H. sapiens*, *Y. pestis* and *R. rattus*) using EAGER (Efficient Ancient GEnome Reconstruction) [41].

sample ID	date	reference genome (data type)	mapped reads after RMDup	mean coverage	genome coverage 1-fold (%)	genome coverage 5-fold (%)	DNA damage 1^st^ base 5'	average fragment length
Rostov2033	1762–1773	*Y. pestis* (enrichment, untrimmed)	868003	12.68	94.48	92.13	0.14	67.99
		*H. sapiens* (shotgun, untrimmed)	157340	0.00	0.30	0.00	0.17	59.84
Rostov2039	1762–1773	*Y. pestis* (enrichment, untrimmed)	226632	4.32	88.40	33.16	0.12	88.80
		*H. sapiens* (shotgun, untrimmed)	248	0.00	0.00	0.00	0.08	76.10
Azov38	15th–17th century	*Y. pestis* (enrichment, untrimmed)	454551	5.18	91.74	52.65	0.16	53.01
		*H. sapiens* (shotgun, untrimmed)	317375	0.01	0.69	0.00	0.13	67.93
Gdansk8	1425–1469	*Y. pestis* (enrichment, untrimmed)	7908081	184.09	95.99	95.81	0.12	108.33
		*H. sapiens* (shotgun, untrimmed)	242174	0.01	0.59	0.00	0.07	84.48
Rat	15th–16th century	*Y. pestis* (enrichment, untrimmed)	913	0.01	1.24	0.01	0.09	73.35
		*R. rattus MT* (shotgun, untrimmed)	2419	9.21	99.78	94.35	0.10	62.07

### (b) Phylogenetic positions of *Y. pestis* from the studied human samples

**Figure 2.**
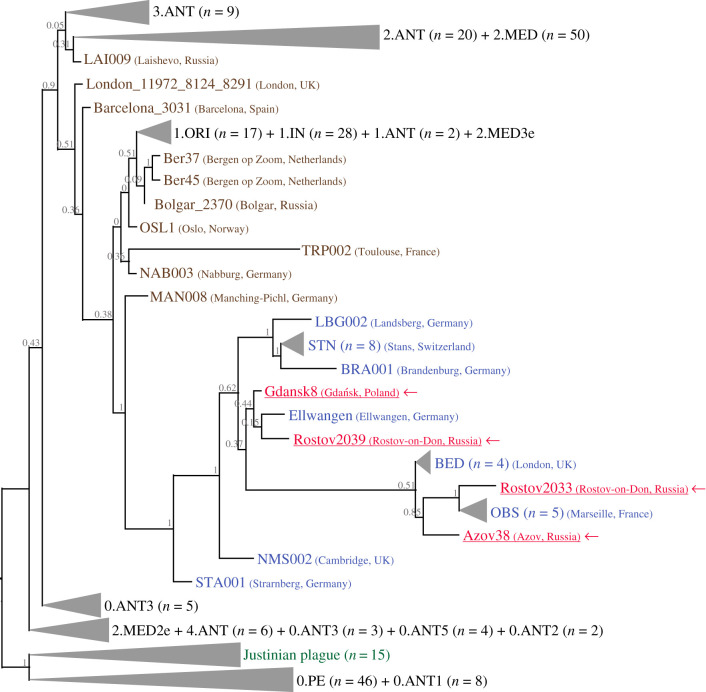
Maximum-likelihood tree (RAxML) showing the location of ancient *Y. pestis* genomes among main *Y. pestis* clusters. The newly studied *Y. pestis* genomes (fifteenth to eighteenth century) are in red and marked by arrows. The previously published samples closer to the Black Death period (thirteenth to fourteenth century, [12,20,27]) are marked in brown. The previously published samples dated to the post-Black Death period (fifteenth to eighteenth century, [18,20,27]) are marked in blue. The samples from the first epidemic (sixth century, [34]) are marked in green. The modern *Y. pestis* strains and first plague epidemic samples are collapsed to improve the tree visibility. The number of samples inside the collapsed branches are indicated in brackets. For detailed information about modern branches, see electronic supplementary material, table S2 and figure S3. Node labels are bootstrap support (100 iterations). *Yersinia pseudotuberculosis* genome [43] was used as an outgroup. For the complete phylogenetic tree, see electronic supplementary material, figure S3. (Online version in colour.)

Maximum-likelihood phylogenetic analysis of our four newly reconstructed *Y. pestis* genomes from Poland (Gdansk8) and Southern Russia (Rostov2033, Rostov2039 and Azov38), together with 257 previously published ancient and modern *Y. pestis* strains [8,9,12,18,20,27,28,32–37,42] (electronic supplementary material, table S2), showed that the newly reconstructed Eastern European strains are located among other medieval and early modern *Y. pestis* strains (figure 2; electronic supplementary material, figure S3). All four newly reconstructed samples are located among post-Black Death genomes including those from France (OBS, eighteenth century), England (BED, sixteenth to seventeenth century), Switzerland (STN, fifteenth to seventeenth century), and Germany (LBG, BRA, Ellwangen, fifteenth to seventeenth century) [18,20] (figure 2; electronic supplementary material, figure S3).

### (c) Functional analysis

Our analysis of SNPs detected within the four new *Y. pestis* genomes from human samples (Gdansk8, Rostov2033, Rostov2039 and Azov38) in comparison to the SNP profiles of previously published ancient *Y. pestis* genomes [1,8,20,27,33,34] revealed that the Southern Russian and Polish samples are characterized by the same spectrum of synonymous and nonsynonymous mutations as other European ancient *Y. pestis* strains (electronic supplementary material, table S4). No unique functional differences were observed that could distinguish East European ancient strains from other ancient ones.

### (d) Molecular analysis of the rat sample

Poor preservation of the highly fragmented rat skull did not allow an analysis of the rat species using morphological methods. Using EAGER [41], the rat shotgun data were mapped against mitochondrial genomes of *Mus musculus*, *Rattus fuscipes*, *Rattus leucopos*, *Rattus norvegicus* and *Rattus rattus* (electronic supplementary material, note S2), which showed that most rat mitochondrial genome reads mapped to *R. rattus* (99% with threefold genome coverage; electronic supplementary material, table S5). In addition, the data were also mapped against the complete nuclear genome of *R. rattus* and *R. norvegicus* (electronic supplementary material, note S2), which confirms the results of the mapping against the mitochondrial genome (electronic supplementary material, table S5). Therefore, the studied rat remains belong, with high probability, to the *R. rattus* (black rat) species. Black rats were hypothesized to be one of the main sources of plague infection in medieval Europe [2,3,21]. As *R. rattus* are absent in Polish territory in the modern period [44], this result, together with a damage profile of 10% (electronic supplementary material, figure S4), supports the authenticity of the rat sample.

The enrichment of the rat sample for *Y. pestis* DNA revealed positive signals. To test the specificity to *Y. pestis*, we mapped the reads from shotgun sequencing and enrichment against other bacteria species from the genus *Yersinia* (*Y*. *enterocolitica*, *Y. pseudotuberculosis*, *Y. similis*, *Y. ruckeri*, *Y. frederiksenii*, *Y. rohdei*, *Y. aldovae*, *Y. intermedia* and *Y. massiliensis*) using MALT [39] (electronic supplementary material, note S2). For other *Yersinia* species, we retrieved 5–235 mapping reads in comparison to 1,618 reads mapped to *Y. pestis* (electronic supplementary material, figure S5). These reads are assigned uniquely to the different species and, in the case of *Y. pestis*, are equally distributed over the genome. Therefore, this observation was the first indication that the obtained reads likely characterize the parts of the *Y. pestis* genome, rather than contamination from other bacteria (electronic supplementary material, figure S5). Subsequently, the sequencing data from shotgun sequencing and enrichment of the rat sample were mapped against the *Y. pestis* genome using EAGER [41] (see electronic supplementary material, note S2 for detailed information). Due to the initial small amount and poor quality of the rat skeletal material, only 1.2% of *Y. pestis* genome was recovered (table 1). The damage profiles (approx. 10%) [40] (electronic supplementary material, figure S4) obtained after mapping against *Y. pestis* (NC_003143.1) and *R. rattus* (NC_012374) genomes showed similar amounts of C > T changes (electronic supplementary material, figure S4). In conclusion, we postulate that the rat likely was infected with *Y. pestis*.

To further specify the positioning of the partially reconstructed *Y. pestis* genome from the rat sample, a maximum-likelihood phylogenetic analysis (electronic supplementary material, figure S6) was conducted also including random strains from different ancient and modern *Y. pestis* branches, as well as *Y. pseudotuberculosis* and *Y. enterocolitica* genomes. The latter two genomes were selected due to their mapping results showing the highest numbers of mapped reads in the rat sample after *Y. pestis* (electronic supplementary material, figure S5). Although the strain could only be partially reconstructed from the rat sample, the identified SNPs are supported by 3–18 reads (electronic supplementary material, table S4). Our analysis places the rat sample into the variety of *Y. pestis* strains, possibly even clustering with ancient *Y. pestis* strains (electronic supplementary material, figure S6); however, due to its low coverage and very low bootstrap support of the tree, more details about its placement among these strains cannot be described.

Interestingly, this rat sample was collected from the same ossuary where the only Polish sample testing positive for plague was found (Gdansk8, electronic supplementary material, table S1). Thus, SNPs identified in the partial *Y. pestis* genome from the rat sample showed differences—however, no functional changes—from those of the human sample (electronic supplementary material, table S4) and the very low bootstrap support of the maximum-likelihood tree did not allow a more detailed analysis of potential connections between these samples (electronic supplementary material, figure S6).

## 4. Discussion

Here, we have reconstructed four genomes of *Y. pestis* from medieval and early historic human samples from Poland and Southern Russia to broaden our knowledge about the genetic diversity of plague circulating in Eastern Europe during the fifteenth to eighteenth century. These new genomes, originating from geographical locations quite distant from previously studied regions, showed the persistence of *Y. pestis* strains phylogenetically close to those previously found in Western Europe [12,18,20,27] and in southeastern regions of Europe.

According to historical records, plague had appeared in what is today northern Poland several times since the fourteenth century [45–47]. In the fifteenth century, it erupted in Gdańsk six or seven times, with the most severe outbreak in 1464 [46,47]. This corresponds well to the ^14^C data for the plague-positive Polish sample Gdansk8 (1425–1469 AD) (table 1; electronic supplementary material, table S1). Before it reached Gdańsk, plague spread in the western European territories, namely the Netherlands, Cologne, Brunswick and Salzburg [46,47]. The phylogenetic proximity of the Gdańsk *Y. pestis* genome, Gdansk8, to other post-Black Death *Y. pestis* strains (figure 2) suggests that the Gdańsk plague epidemics were included in the waves affecting Western Europe during the fifteenth to eighteenth century.

We see a similar picture with the *Y. pestis* strains from Southern Russia. The most probable source of the eighteenth century plague epidemic in Southern Russia likely relates to Russian soldiers returning after the Russo-Turkish War of 1768–1774 [26,48]. This plague epidemic was the last severe outbreak in Europe [26,48]. It spread widely and was the cause of the Moscow plague riot of 1771 [48]. While the phylogenetic position of two eighteenth century Southern Russian *Y. pestis* genomes, Rostov2033 and Rostov2039, among Western European post-Black Death strains (figure 2) does not confirm or refute the Turkish origin of the pathogen, it supports the relation of Southern Russian eighteenth century plague with the Western European epidemics. However, it is worth noting that the Rostov-on-Don region was located on the crossroads of multiple water and land routes, and plague strains could have been brought into this region via many different ways [48]. This assumption is supported by the position of the two Rostov samples on the phylogenetic tree: despite the fact they originated from the same burial ground, they do not cluster together (figure 2).

Overall, potential causes of the fifteenth to seventeenth century plague in Southern Russia are not well known as both external (i.e. European, through Ukraine or Crimea, or Asian, probably Persian) and internal (some residual natural) reservoirs are hypothesized [49]. The location of Azov38 (fifteenth to seventeenth century) *Y. pestis* genome close to other European *Y. pestis* strains suggests some external origin, but in the absence of ancient *Y. pestis* genomes from Asia, the exact source (i.e. western or eastern) of these plague strains cannot yet be identified.

Furthering the work done by other researchers [8,9,14,20], we also performed a phylogenetic time-scale reconstruction including our four new *Y. pestis* genomes (see electronic supplementary material, note S3, figures S7 and S8). Our estimated origin age of Branch 1 *ca.* 1270 AD is similar to the age estimated by Spyrou and colleagues [20] (see electronic supplementary material, note S3 and figure S8). However, due to the known issues with highly variable nucleotide substitution rates among *Y. pestis* strains affecting the credibility of both topology and time-scale estimates [8], reconstruction of genealogical trees for plague remains controversial [8]. Therefore, we provide our time-scale estimation as provisional guidelines into the timing of *Y. pestis* lineage splits (electronic supplementary material, figure S8), which may prove a useful reference for future research but should be interpreted with caution.

Next, it is further worth examining the strain diversity we found among Eastern European *Y. pestis* (figure 2; electronic supplementary material, figure S3). Two genetically different *Y. pestis* strains coexist in one Rostov-on-Don burial ground (Rostov2033 and Rostov2039; figure 2). Interestingly, the previously published data on medieval *Y. pestis* genomes from another Russian region, Tatarstan [20,27], shows a similar picture: two different *Y. pestis* strains (LAI009 and Bolgar 2370) coexist within a relatively small territory (i.e. the distance between the two Tatarstan burial grounds, Laishevo and Bolgar, is 140 km) during the same time period (fourteenth century) (figure 2 in our study; fig. 2 in Spyrou *et al.* [20]). In both cases (Rostov-on-Don and Tatarstan), the time synchronism of the samples does not suggest ancestor–descendant relationships between the strains, but rather may point to several different introductions of *Y. pestis* in Eastern Europe or the existence of different local reservoirs. It is of note that the complex picture of the genetic diversity of *Y. pestis* Black Death strains is observed not only in Eastern, but also Western Europe. Indeed, the post-Black Death samples (fifteenth to eighteenth century) form a rather compact cluster (figure 2); at the same time, the Black Death samples (thirteenth to fourteenth century) do not cluster together (figure 2; electronic supplementary material, figure S3), which indicates higher genetic diversity. Taken together, this may suggest either several independent plague introductions or the existence of different plague reservoirs in Eastern Europe during the medieval and early modern era. Such scenarios could be further supported by observations of climate fluctuations in Europe providing opportunities for repeated climate-driven reintroductions of *Y. pestis* into Europe from various animal reservoirs [15].

Plague reservoirs in medieval Europe are poorly documented, and mechanisms of plague transmission to humans in the past are still actively discussed [24,25,50]. Black rats, also known as ship rats, were considered by many researchers as the main source of plague in Europe during the first and the second epidemics, and their replacement by brown rats in the nineteenth century was believed to be the reason for the decline of the Black Death [51,52]. However, this theory has been highly debated due to the discordance between the dates of the reduction of black rat population and waning of the disease [53]. Additionally, the rapid spread of plague during the Black Death led researchers to suppose other means of plague transmission [25]. Our partial reconstruction of the *Y. pestis* genome obtained from rat remains collected at a Polish ossuary, dating to the fifteenth to sixteenth century, is the first genetic contribution to this debate. However, we acknowledge limitations surrounding our data. Despite the limited reference data for rat species on the mitochondrial and nuclear level (i.e. two nuclear genomes and 16 mitochondrial genomes retrievable from GenBank [38]), we can link our rat sample with a high probability to *R. rattus;* however, we cannot fully exclude potential similarities to rat species which have yet to be sequenced. Although the poor DNA preservation of the partial *Y. pestis* genome did not allow for a well-resolved placement in the phylogenetic tree to determine its exact position among *Y. pestis* strains (electronic supplementary material, figure S6), we can for the first time—to our best knowledge—link *Y. pestis* DNA with medieval animal remains, and very likely with black rats in particular. Thus, we cannot detect an ongoing transmission to humans in the medieval era due to SNP differences of the Gdansk8 genome and the rat *Y. pestis* partial genome (electronic supplementary material, table S4) as well as the low resolution of rat *Y. pestis* in the phylogenetic tree (electronic supplementary material, figure S6). In conclusion, our results provide new information concerning potential natural plague sources in Europe, suggesting that black rats were at least one possible source of plague distribution in medieval Europe.

Overall, our new data demonstrates the importance of adding information from historical Eastern European *Y. pestis* strains to construct a more comprehensive picture of *Y. pestis* diversity in Europe. Based on our observations, we expect the genetic diversity of plague bacteria in Europe, especially of the strains dated closer to the Black Death period (thirteenth to fourteenth century), to be more complex. Further sampling from different Eastern European and Western Asian regions, as well as additional ancient *Y. pestis* genomes from potential animal plague carriers, stand to add more complexity and will deepen our knowledge regarding plague sources and its transmission in Europe.

## Supplementary Material

Supplementary notes, figures and tables

## Supplementary Material

Supplementary Table 4

## Data accessibility

The raw sequencing data have been deposited at the European Nucleotide Archive (ENA) under project accession ID PRJEB35426.
