# Supplementary material for: New ancient Eastern European Yersinia pestis genomes illuminate the dispersal of plague in Europe
Source: Philos Trans R Soc Lond B Biol Sci. 2020 Oct 5;375(1812):20190569. doi: 10.1098/rstb.2019.0569 (PMC7702796; doi:10.1098/rstb.2019.0569)
Supplement: Supplementary notes, figures and tables [file rstb20190569supp1.pdf]

SUPPLEMENTARY INFORMATION

New ancient Eastern European *Yersinia pestis* genomes illuminate the dispersal of plague in Europe

Irina Morozova, Artem Kasianov, Sergey Bruskin, Judith Neukamm, Martyna Molak, Elena Batieva, Aleksandra Pudło, Frank J. Rühli, Verena J. Schuenemann

Supplementary Note 1: Archaeological background of the samples

The cemetery of St. Dmitry Rostovsky fortress (Rostov-on-Don, Russia)

The cemetery of St. Dmitry Rostovsky fortress was excavated in 1998-2004. The cemetery was divided into several segments, with most burials containing multiple individuals. Two of these collective burials had traces of lime present, indicating disinfection of the corpses was likely performed [1-3]. Burial rites were characterized as Christian and have been dated to the second half of the 18th century. A section of the burials was dated more precisely to 1762-1773 AD (Supplementary Table 1). Documentary sources provided a means to connect at least a few of the burials with plague epidemics in Rostov-on-Don in the winter of 1771 (Elena Batieva, personal communication). Teeth from 39 individuals were analyzed from several sectors of the cemetery (Supplementary Table 1). The samples were provided under the Agreement on Scientific collaboration with Southern Scientific Center, Russian Academy of Sciences.

Azov city (Rostov-on-Don region, Russia)

Skeletal remains were discovered in 2005-2012 during a rescue excavation in the historical city of Azov located southwest of Rostov-on-Don. Burial artifacts and stratigraphy date the remains to the 15th-18th centuries, after the fall of the Golden Horde (Elena Batieva, personal communication). Four samples were analyzed during this study (Supplementary Table 1). The samples were provided under the Agreement on Scientific collaboration with Southern Scientific Center, Russian Academy of Sciences.

Gdańsk ossuaries (Gdańsk, Poland)

The three ossuaries were discovered during archaeological excavations in the Dominican Square in 2009–2011 [4]. Based on the stratigraphy and burial artifacts, the ossuaries were dated to the 15th-18th centuries [5]. In general, the skeletal material in the ossuaries was intermingled and bones were disarticulated. In one of these ossuaries, Ossuarium 3009, significant amounts of lime were found, suggesting a connection to plague epidemics. Teeth from 35 individuals from three ossuaries were analyzed. One *pla*-positive sample was discovered during the preliminary screening [6]. Additionally, three skull fragments (about one square cm each) from a rat found in the Ossuarium 3009 (15th-16th century) were also sampled for DNA analyses (Supplementary Table 1). For the extraction step, samples from these fragments were combined and extracted together and is referred to the rat sample in this study. All samples from Gdańsk were provided under the Agreement on Scientific collaboration with the Archaeological Museum in Gdańsk.

For the *pla*-positive human sample, ^{14}C dating analysis was performed at the Laboratory of Ion Beam Physics, ETH Zurich (lab number ETH-101916). The sample was dated to 1425-1469 AD (^{14}C age BP (1950) 443), which corroborates the archaeological data.

Supplementary Note 2: Methods

DNA extraction

DNA was extracted at the Paleogenetics laboratory at the Institute of Evolutionary Medicine, University of Zurich, Switzerland. Both the laboratory equipment and experimental protocols conform to all requirements for ancient DNA studies [7, 8]. Ancient DNA work was performed with all the precautionary measures needed to prevent contamination: regular UV-treatment and chemical sterilization of all working surfaces and reusable instruments; use of sterile disposable clothes, sterile filtered tips etc.; negative controls were included at each stage of the experiment and carried through.

DNA was extracted using the protocol described by Rogaev and colleagues [9], with minor modifications. Briefly, 0.2–1.0 g of the sample was decontaminated (mechanical removing of the surface using handheld drill (Proxxon), UV irradiation for 15 min), powdered (Cryogenic

SpexMill), decalcified in lysis buffer (0.5 M EDTA, 10% proteinase K), and incubated with rotation for 48 hours at 37°C. The bone powder was then precipitated by centrifugation for 5 min at maximum speed, the supernatant was concentrated using Amicon centrifugal units (30 kD, Millipore) to the final volume 100–150 µl. DNA was then extracted from the filtrate using silica spin columns (Qiagen MinElute PCR Purification Kit) according to the manufacturer's protocol. The final volume of the extract was 60 µl. DNA quantity was assessed using Qubit fluorometer (Thermo Fisher Scientific). DNA extracts were stored at –20°C.

Primary screening

Initial screening for the presence of *Y. pestis* DNA was performed using primers specific to the plasminogen activator (*pla*) gene located on the high-copy pPCP1 plasmid of *Y. pestis* as described elsewhere (52 bp fragments, [10]). In addition, the primers for longer *pla*-fragments (133 bp fragments, [11]) and subsequent Sanger sequencing was done, in order to exclude false-positive results. As a control for the presence of bacterial DNA of the same length in the extracts, a parallel PCR was performed using universal primers for V6 region of bacterial 16S rRNA [12]. *Pla*-positive samples were built into NGS libraries [13, 14]. Due to a small amount of skeletal material for the rat, the screening stage for the rat sample was skipped, and the rat DNA was directly transformed into NGS libraries following the same methods [13, 14].

NGS Library preparation and shotgun sequencing

Double-stranded indexed Illumina libraries were constructed according to the protocols [13, 14] specifically developed for ancient DNA. Index combinations containing unique 8 bp barcodes were used for double indexing. Ten PCR cycles were used for the indexing step. Indexed libraries were quantified using Agilent 2200 TapeStation System, and equimolar quantities of every library were pooled together and sequenced on Illumina HighSeq 4000 with 2*75+8+8 cycles. The sequencing was performed at the Functional Genomics Center Zurich, Switzerland.

Target enrichment

The five samples showing positive *Y. pestis* signals in shotgun sequencing (Rostov16039, Rostov2033, Rostov2039, Azov38, and Gdansk8) and the rat sample were subjected to target enrichment [15, 16]. The libraries prepared for shotgun sequencing were used for enrichment.

SeqCap EZ Prime Developer Probes (Roche) were used for in-solution capture. Full *Y. pestis* chromosome (NC_003143.1) and three plasmids, pCD1 (NC_003131.1), pMT1 (NC_003134.1), and pPCP1 (NC_003132.1) were used to design the probes. Target enrichment was performed according to the manufacturer's protocol. Briefly, amplified indexed libraries were mixed in equimolar amounts to a final concentration of 1.5 µg of DNA per capture reaction and hybridized with DNA capture probes using the following regimen: 95°C for 5 min, 47°C for 20 hrs in a thermocycler with heated lid (57°C). After this, the captured DNA samples were washed using HyperCap Beads (Roche). The whole bead-bound DNA samples (about 20 µl) were used for subsequent PCR amplification with the same pair of primers which was used for amplification of indexed DNA libraries prior to enrichment. 14 cycles of amplification were performed. Enriched libraries were quantified using Agilent 2200 TapeStation System and sequenced on Illumina NextSeq500 with 2*75+8+8 cycles (Functional Genomics Center Zurich).

Data analysis

Read processing, mapping, and variant calling

First, all libraries belonging to the same individual were merged. Then, all samples were processed using EAGER version 1.92.55 [17]. To summarize, the sequencing quality was inspected with FastQC version 0.11.5 [18], the reads were adapter trimmed and read pairs merged with AdapterRemoval version 2.2.1a [19] and subsequently aligned to the *Y. pestis* CO92 chromosome (NC_003143.1) using CircularMapper version 1.0 [17] with a minimum quality score of 37 and a maximum edit distance of n=0.01. Duplicates were removed with MarkDuplicates version 2.15.0 (Picard Tools - By Broad Institute, n.d.), and DamageProfiler version 0.3.12 [20] was used to investigate the damage patterns.

Before variant calling, one base at the 5' and 3' end, respectively, was trimmed by one base pair using FASTX-Toolkit (http://hannonlab.cshl.edu/fastx_toolkit/) to remove sites that could have been affected by ancient DNA damage. Subsequently, the reads were re-filtered for length and remapped using the parameters described above. The Genome Analysis Toolkit (GATK) version 3.8.0 [21, 22] was used to generate a mapping assembly and SNP calling. The reference base was called if the position was covered by a read at least three times and the quality score was at least 30. The base was called as a SNP if the quality score was at least 30 and 90% of the mapped reads contained this variant.

In addition, all merged libraries from humans were mapped to the human mitochondrial genome (NC_012920.1) as described above with the exception of variant calling.

To assign the rat to the correct species, this sample was independently mapped against different reference mitochondrial genomes of the genus *Rattus*, namely *Rattus fuscipes* (NC_014867.1), *Rattus leucopos* (NC_014855.1), *Rattus norvegicus* (NC_001665.2), and *Rattus rattus* (NC_012374.1) using the parameters described when mapping against *Y. pestis*, with the exception of the variant calling. In addition, the sample was mapped against *Mus musculus* (NC_005089.1). Furthermore, these data were mapped to the complete nuclear genome of *R. rattus* and *R. norvegicus*, which are the only complete nuclear genomes available for this genus. For this purpose, BWA aln [23], instead of CircularMapper [17], was used for mapping.

Constructing SNP alignment

The four newly reconstructed and 257 previously published ancient and modern *Y. pestis* genomes ([2, 15, 24-35], Supplementary Table 2) were used for phylogenetic reconstruction. We only included samples that fulfilled quality criteria of at least 3fold coverage at each called site and at least 60% of the reference genome covered. Also, the strains SCL1006, NAB005, STN011, STN004, NAB005, and BRA003 were excluded due to possible environmental contamination [34]. In addition, we excluded strain TRP002 as it is likely contaminated [27]. All published strains were treated with the EAGER pipeline [16] as described above. For genomes where only the fasta sequence was available, sequencing reads were simulated using Genome2Reads (https://github.com/shendurelab/HybridYeastHiC).

Metagenomic screening

To detect the presence of *Y. pestis* in studied samples and determine the *Yersinia* species that is most likely present in the rat sample, we performed a comparative mapping with MALT [36] using all complete bacterial, viral, and archaeal genomes in GenBank [37] as a reference (version May 2018). MALT was executed with the following mapping parameters: Only reads with a minimum 85% identity (--minPercentIdentity) were considered as a possible match to the reference. Moreover, the minimum support parameter (--minSupport) was set to 5, i.e. only nodes with minimum support of five reads are kept. BlastN mode and SemiGlobal alignment were applied and

a top percent value (--topPercent) of 1 was set. All other parameters were set to default. MALT results were analyzed and visualized using MEGAN6 [38].

The reference database also includes various *Yersinia* strains, which were used for the identification of the reads mapping to *Yersinia* from the rat sample (*Y. enterocolitica* (NC_008800.1), *Y. pseudotuberculosis* (NC_010634.1), *Y. similis* (NZ_CP007230.1), *Y. ruckeri* (NZ_CP011078.1), *Y. frederiksenii* (NZ_CP009364.1), *Y. rohdei* (NZ_CP009787.1), *Y. aldovae* (NZ_CP009781.1), *Y. intermedia* (NZ_CP009801.1), and *Y. massiliensis* (NZ_CP028487.1)).

Phylogenetic tree reconstruction

Four newly reconstructed and 257 previously published ancient and modern *Y. pestis* genomes ([2, 15, 24-35], Supplementary Table 2) were used for phylogenetic reconstruction. For creating consensus sequences, bcftools version 1.7 (http://www.htslib.org/doc/bcftools-1.7.html) was used. Indels were excluded from vcf files before creating consensus sequences. The regions with coverage below three were masked during consensus construction. Next, CDS sequences were extracted from consensus sequences using gffread software from GFF Utilities version 0.11.5 (http://ccb.jhu.edu/software/stringtie/gff.shtml). Concatenation of CDS sequences was used in phylogenetic tree reconstruction using RaXML software version 8.2.4 [39] with parameters "-m GTRCAT -x 123456 -N 100 -p 098765" and outgroup *Y. pseudotuberculosis* IP32953.' In the analysis, 100 bootstrap iterations were used.

To access the phylogenetic placement of the partial *Yersinia pestis* strain reconstructed from the rat sample, a maximum likelihood tree was calculated based on a SNP alignment using positions that were covered at least three times. We added one random strain per branch, the rat strain, *Y. pseudotuberculosis*, and *Y. enterocolitica* since they two contained the maximum number of mapped reads after *Y. pestis* (Supplementary Figure 5). The alignment was created as described above. RAxML version 8.2.12 [39] was used with 100 bootstraps and the GTR - GAMMA model.

BEAST analysis

We used the Bayesian framework BEAST v1.10.4 [40] to estimate divergence times and substitution rates. All published modern and ancient strains [2, 15, 24-35] were treated with the EAGER pipeline [17] using the parameters described above. In the analysis, we only included the samples representing branch 1 of the *Y. pestis* phylogeny that fulfilled quality criteria of at least

3fold coverage at each called site and at least 60% of the reference genome covered. The SNP alignment was built with MUSIAL (https://github.com/Integrative-Transcriptomics/MUSIAL) and a SNP was used when it was called in at least one sample. All positions with more than 3% missing data were excluded. The resulting SNP alignment consisted of 620 SNPs for a total of 82 historical and modern strains [15, 25, 27, 28]. No outgroup sequence was included in compliance with BEAST Bayesian dated phylogeny assumptions.

The GTR nucleotide substitution model was used according to ModelGenerator version 851 [41] analysis applying Bayesian Information criterion. A relaxed uncorrelated log-normal clock with CTMC Rate Reference prior and Bayesian skyline tree model were used. The MCMC chain was run for 300 million steps with sampling every 10,000th step. Convergence and mixing were inspected in Tracer v1.7.1 [42] with all ESS exceeding value 100. The Maximum Clade Credibility tree was built using TreeAnnotator (part of BEAST package) and visualized using FigTree v1.4.2 (http://tree.bio.ed.ac.uk/software/figtree/).

Temporal signal in the dataset was investigated using Date-Randomisation Test (DRT, [43]; prepared using an unpublished R script by Sebastian Duchene) and Bayesian Evaluation of Temporal Signal (BETS, [44]). In DRT, substitution rate estimates for ten replicates of the BEAST analysis with tip dates randomized among the samples do not overlap with the estimate using the original tip dates (Supplementary Figure 7), indicating sufficient temporal signal for calibrated phylogenetic analysis in the dataset. BETS analysis using Bayes Factor based model selection also detected temporal signal supporting the justification for our tip dating analysis with Bayes Factors of $1.97x10^{96}$ and $2.65\ x10^{96}$ to support the tip dated versus isochronous phylogeny using path sampling and stepping stone sampling, respectively. Root-to-tip regression was projected using TempEst v1.5.3 [45]; however, it did not support the presence of temporal signal in the data resulting in Rsq = 0.19 and a Correlation Coefficient of -0.43.

Genome coverage

Per base depth was obtained by using samtools depth software. CG count were counted in 100 bp windows for *Y. pestis* CO92 genome. Circular graphs for depth and GC content were created using CIRCOS software [46]. Full *Y. pestis* chromosome (NC_003143.1) and three plasmids, pCD1 (NC_003131.1), pMT1 (NC_003134.1), and pPCP1 (NC_003132.1) were used for the analysis.

Functional analysis

A dataset of 37 ancient *Y. pestis* genomes (five newly reconstructed and 32 previously published ancient strains [15, 25, 27-30], Supplementary Table 2) was functionally annotated using SnpEff [47] version 4.3t.

Supplementary Note 3: Results phylogenetic timescale reconstruction

Despite the likely issues with phylogenetic dating plague [25], we performed a dated phylogeny estimation similar to previous studies (e.g. Spyrou and colleagues [27]) to explore the possible timescale of *Y. pestis* evolution with the newly generated genetic data. Our Bayesian dated phylogeny for Branch 1 (Supplementary Figure 8) revealed the origin of the branch ~740 years ago (95% CI 670 – 940 years ago), i.e. ca.1270 AD, similar to the age estimated by Spyrou and colleagues [27]. Posterior probability varies highly throughout the tree, with very low support values for the nodes comprising Black Death samples. It was previously indicated that the substitution rates vary drastically among different *Y. pestis* lineages [25]. Therefore, the reconstruction of an uncontroversial genealogical tree seems difficult [25]. Even though our analyses suggest sufficient temporal signal in the dataset for the timescale reconstruction (Supplementary Figure 7 and Supplementary Table 6), we thus urge the readers to interpret the time estimates shown in the tree with caution and as due for further confirmation with future research.

The low support values for certain nodes could also be caused by the absence of sufficient data from eastern regions (i.e. Eastern Europe and Asia). Thus, we can expect that the location of some ancient *Y. pestis* samples on the tree may be changed with the addition of new data. These two reasons, as well as the higher quality thresholds for the sequences included in the BEAST analysis which resulted in a much smaller (both lengthwise and in the number of samples included) alignment as compared to the one used for the ML phylogeny, could explain the discrepancies between the ML and BEAST trees (Figure 2 and Supplementary Figure 8).

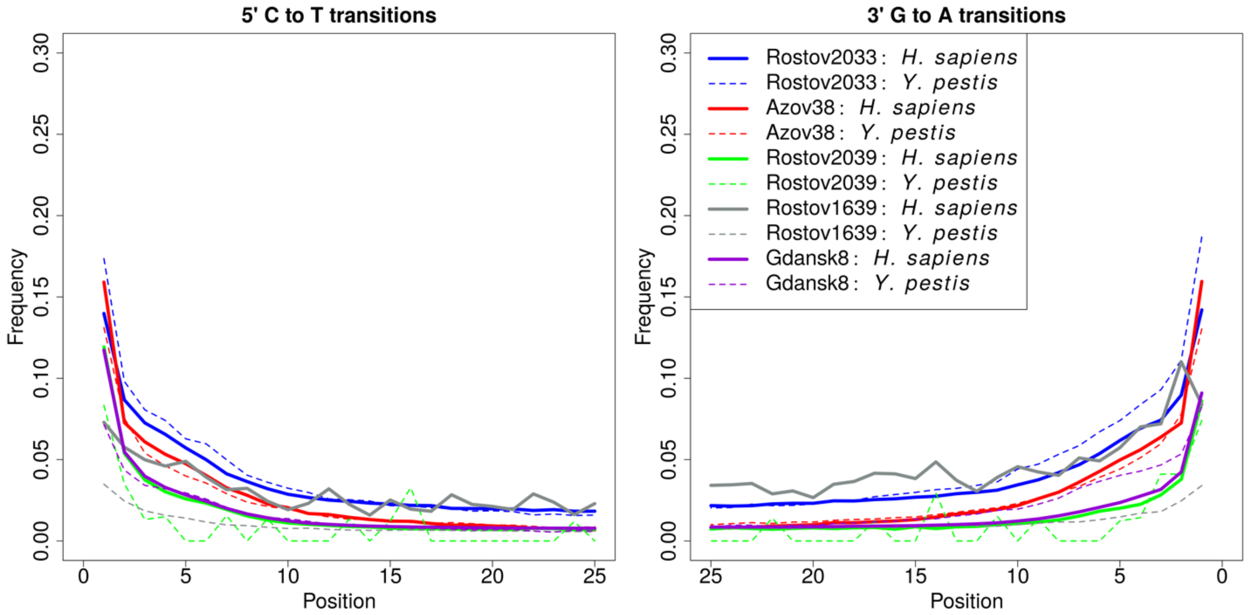

Supplementary Figure 1. Damage profiles of the studied human samples. Damage profiles for samples Rostov2033, Rostov2039, Rostov1639, Azov38, and Gdansk8. Alignments against *Y. pestis* are in dashed lines, those against *H. sapiens* are in solid lines.

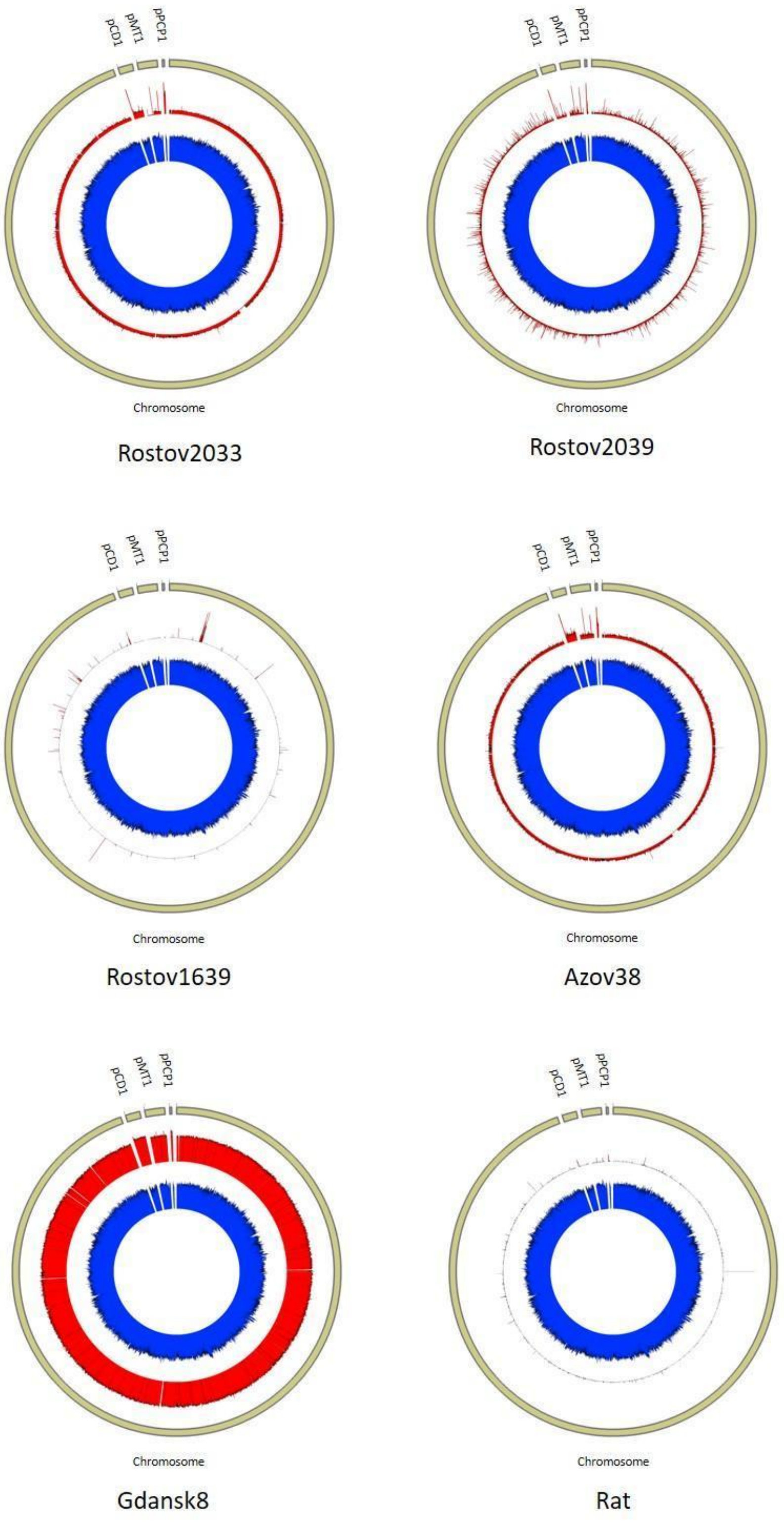

Supplementary Figure 2. Genome coverage after enrichment for *Y. pestis*. The coverage is in red, GC content is in blue. Since the GC content was calculated using a sliding window, it is shown even for uncovered regions. For details, see Supplementary Note 2.

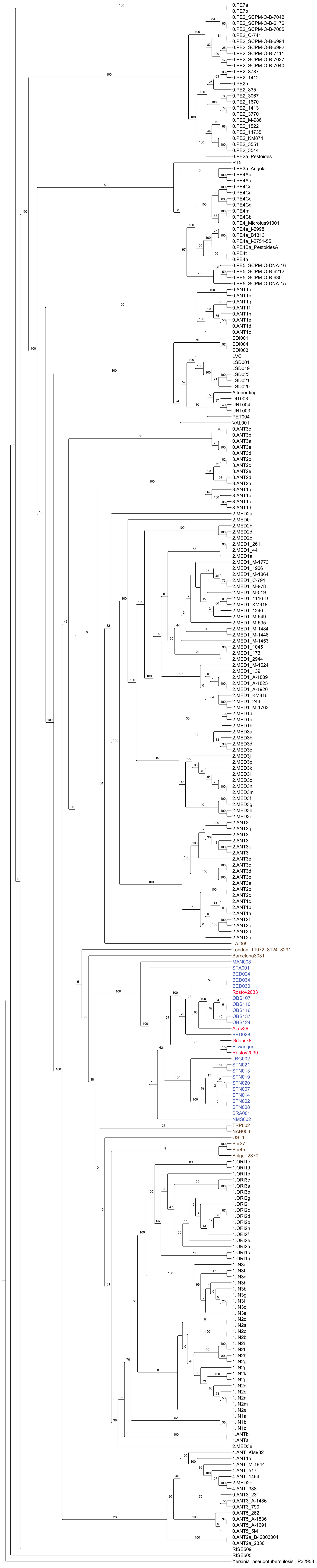
0.PE7a
0.PE7b
0.PE2_SCPM-O-B-7042
0.PE2_SCPM-O-B-6176
0.PE2_SCPM-O-B-7005
0.PE2_C-741
0.PE2_SCPM-O-B-6994
0.PE2_SCPM-O-B-6992
0.PE2_SCPM-O-B-7111
0.PE2_SCPM-O-B-7037
0.PE2_SCPM-O-B-7040
0.PE2_8787
0.PE2_1412
0.PE2b
0.PE2_835
0.PE2_3067
0.PE2_1670
0.PE2_1413
0.PE2_3770
0.PE2_M-986
0.PE2_1522
0.PE2_14735
0.PE2_KM874
0.PE2_3551
0.PE2_3544
0.PE2a_Pestoides
RT5
0.PE3a_Angola
0.PE4Ab
0.PE4Aa
0.PE4Cc
0.PE4Ca
0.PE4Ce
0.PE4Cd
0.PE4m
0.PE4Cb
0.PE4_Microtus91001
0.PE4a_I-2998
0.PE4a_B1313
0.PE4a_I-2751-55
0.PE4Ba_PestoidesA
0.PE4t
0.PE4h
0.PE5_SCPM-O-DNA-16
0.PE5_SCPM-O-B-6212
0.PE5_SCPM-O-B-630
0.PE5_SCPM-O-DNA-15
0.ANT1a
0.ANT1b
0.ANT1g
0.ANT1f
0.ANT1h
0.ANT1e
0.ANT1d
0.ANT1c
EDI001
EDI004
EDI003
LVC
LSD001
LSD019
LSD023
LSD021
LSD020
Altenerding
DIT003
UNT004
UNT003
PET004
VAL001
0.ANT3c
0.ANT3b
0.ANT3a
0.ANT3e
0.ANT3d
3.ANT2b
3.ANT2c
3.ANT2e
3.ANT2d
3.ANT2a
3.ANT1a
3.ANT1b
3.ANT1c
3.ANT1d
2.MED2a
2.MED0
2.MED2b
2.MED2d
2.MED2c
2.MED1_261
2.MED1_44
2.MED1a
2.MED1_M-1773
2.MED1_1906
2.MED1_M-1864
2.MED1_C-791
2.MED1_M-978
2.MED1_M-519
2.MED1_1116-D
2.MED1_KM918
2.MED1_1240
2.MED1_M-549
2.MED1_M-595
2.MED1_M-1484
2.MED1_M-1448
2.MED1_M-1453
2.MED1_1045
2.MED1_173
2.MED1_2944
2.MED1_M-1524
2.MED1_139
2.MED1_A-1809
2.MED1_A-1825
2.MED1_A-1920
2.MED1_KM816
2.MED1_244
2.MED1_M-1763
2.MED1d
2.MED1c
2.MED1b
2.MED3a
2.MED3b
2.MED3d
2.MED3c
2.MED3j
2.MED3p
2.MED3k
2.MED3l
2.MED3o
2.MED3n
2.MED3m
2.MED3f
2.MED3g
2.MED3h
2.MED3i
2.ANT3i
2.ANT3g
2.ANT3j
2.ANT3
2.ANT3k
2.ANT3l
2.ANT3e
2.ANT3c
2.ANT3d
2.ANT3b
2.ANT3a
2.ANT2b
2.ANT2c
2.ANT1c
2.ANT1b
2.ANT1a
2.ANT2f
2.ANT2e
2.ANT2d
2.ANT2a
LAI009
London_11972_8124_8291
Barcelona3031
MAN008
STA001
BED024
BED034
BED030
Rostov2033
OBS107
OBS110
OBS116
OBS137
OBS124
Azov38
BED028
Gdansk8
Ellwangen
Rostov2039
LBG002
STN021
STN013
STN019
STN020
STN007
STN014
STN002
STN008
BRA001
NMS002
TRP002
NAB003
OSL1
Ber37
Ber45
Bolgar_2370
1.ORI1e
1.ORI1d
1.ORI1b
1.ORI3c
1.ORI3a
1.ORI3b
1.ORI2g
1.ORI2i
1.ORI2c
1.ORI2d
1.ORI2b
1.ORI2h
1.ORI2f
1.ORI2e
1.ORI2a
1.ORI1c
1.ORI1a
1.IN3a
1.IN3f
1.IN3d
1.IN3h
1.IN3b
1.IN3g
1.IN3i
1.IN3c
1.IN3e
1.IN2d
1.IN2a
1.IN2c
1.IN2b
1.IN2i
1.IN2f
1.IN2h
1.IN2g
1.IN2p
1.IN2k
1.IN2j
1.IN2q
1.IN2o
1.IN2n
1.IN2m
1.IN2e
1.IN1a
1.IN1b
1.IN1c
1.ANTb
1.ANTa
2.MED3e
4.ANT_KM932
4.ANT1a
4.ANT_M-1944
4.ANT_517
4.ANT_1454
2.MED2e
4.ANT_338
0.ANT3_231
0.ANT3_A-1486
0.ANT3_790
0.ANT5_262
0.ANT5_A-1836
0.ANT5_A-1691
0.ANT5_5M
0.ANT2a_B42003004
0.ANT2a_2330
RISE509
RISE505
Yersinia_pseudotuberculosis_IP32953

Supplementary Figure 3. Phylogenetic relationships between ancient and modern *Y. pestis* strains. Maximum Likelihood tree was constructed based on four newly reconstructed genomes (Rostov2033, Rostov2039, Azov38, and Gdansk8) and 257 previously published ancient and modern *Y. pestis* genomes ([2, 15, 24-35], Supplementary Table 2). *Y. pseudotuberculosis* genome [48] was used as an outgroup. The newly studied *Y. pestis* genomes (15-18th centuries) are colored in red; the previously published samples dating to the Black Death period (13-14th centuries, [15, 27, 28]) are in brown. Previously published samples dated to the post-Black Death period (15-18th centuries, [24, 27]) are marked in blue.

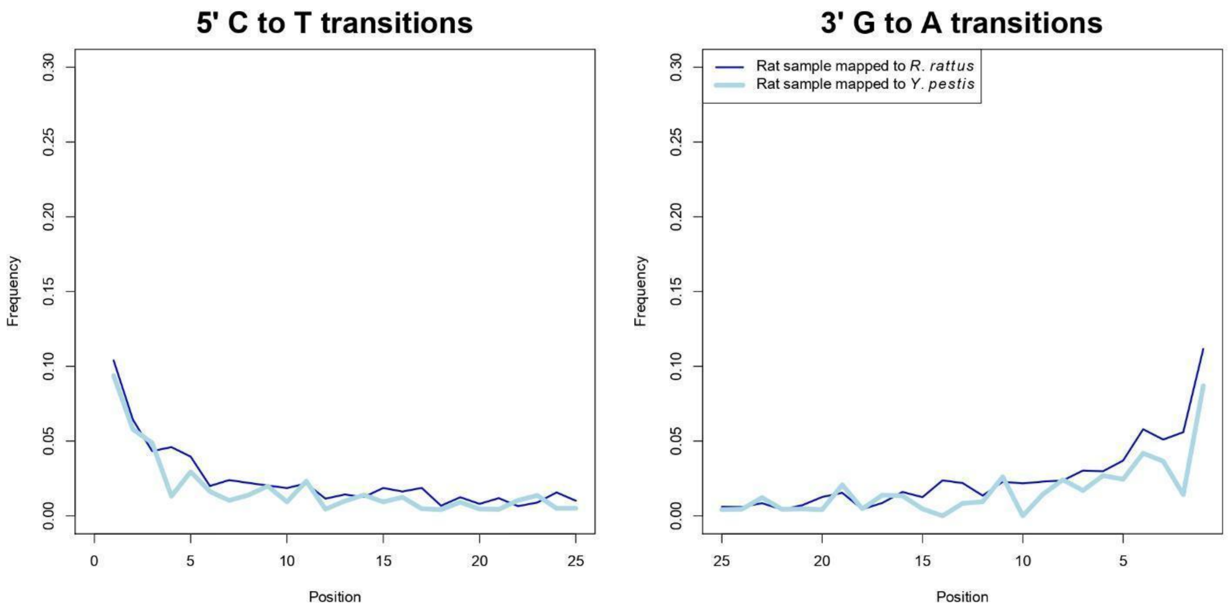

Supplementary Figure 4. Damage profiles of the rat sample. Alignment to *Y. pestis* is depicted in light-blue, to *R. rattus* is in blue.

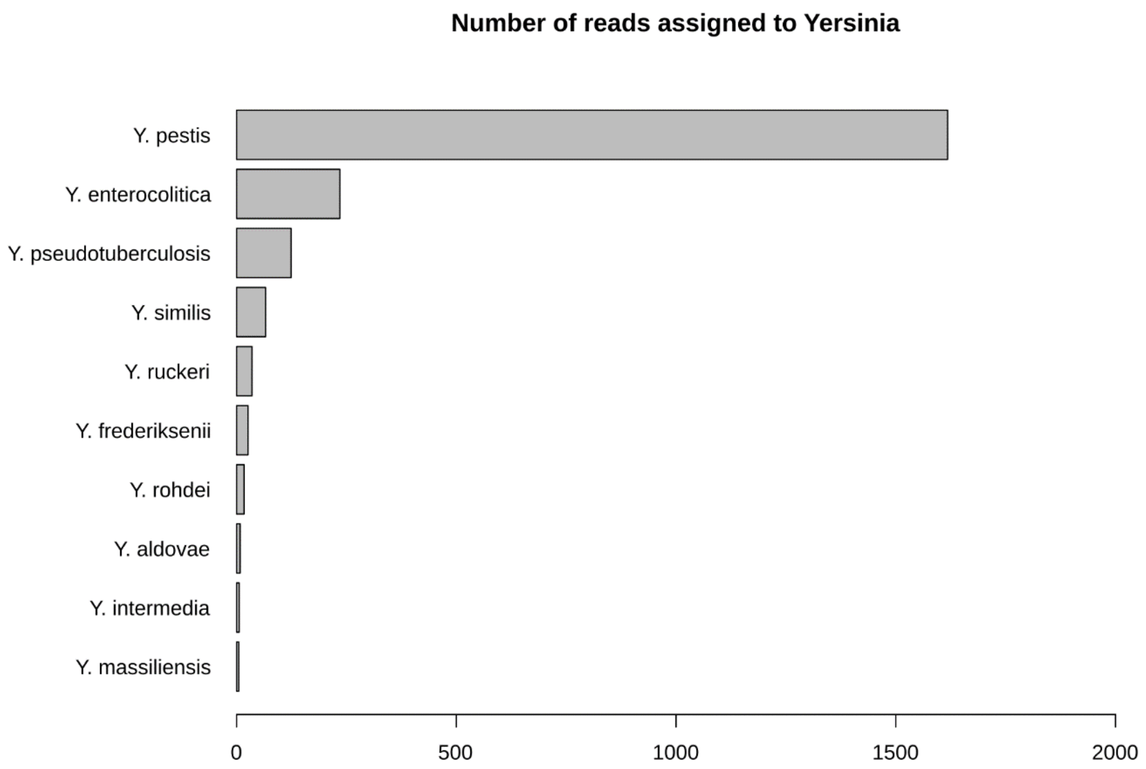

Supplementary Figure 5. Mapping the rat sample against different Yersinia species. The number of reads uniquely assigned to the different *Yersinia* species. MALT [36], with all complete bacterial, viral, and archaeal genomes in GenBank [37] as a reference (version May 2018), was used for comparative mapping. In total, 3,784 reads mapped on genus-level. This also includes measured values that deviated from the species-level because of the same mapping probability.

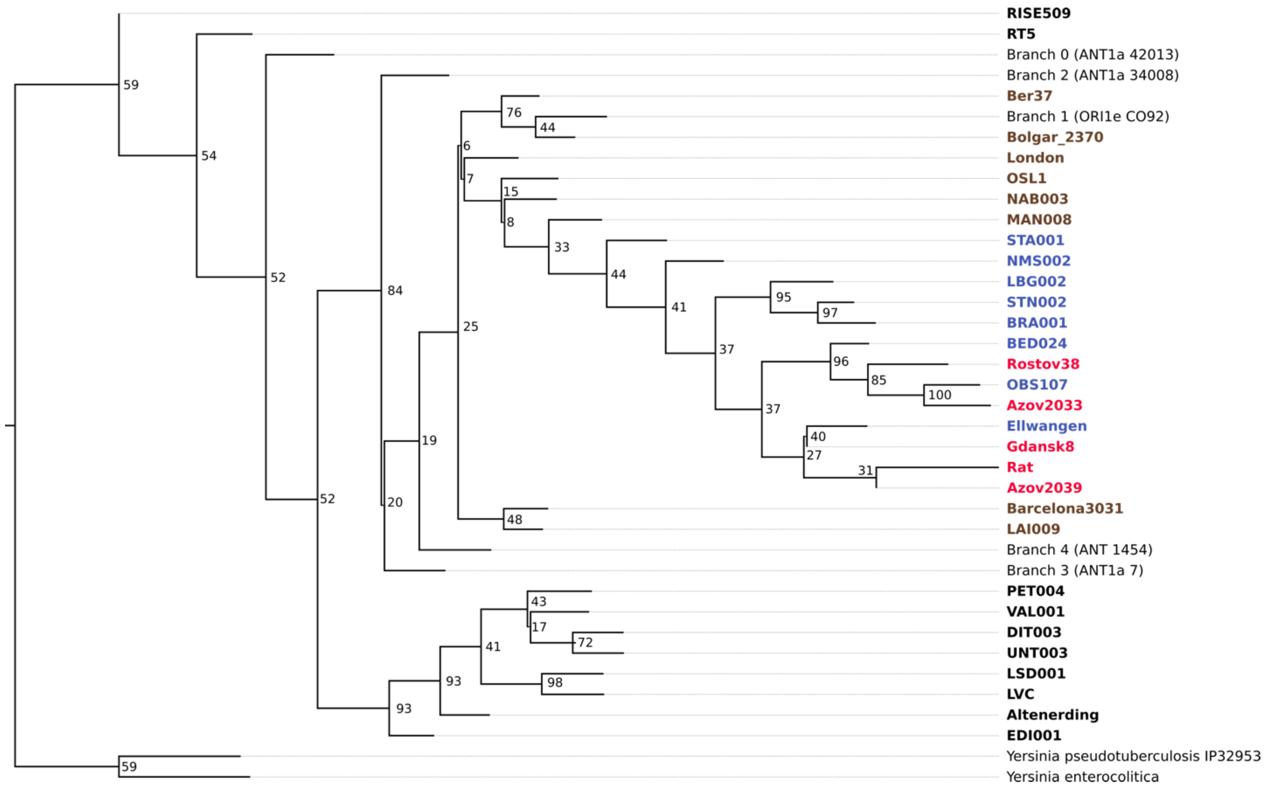

Supplementary Figure 6. Phylogenetic (ML) location of the rat *Y. pestis* partially reconstructed genome among random ancient and modern Yersinia strains. Colors are similar to Supplementary Figure 3: The newly studied *Y. pestis* genomes (15-18th centuries) are in red; previously published samples dating to the Black Death period (13-14th centuries, [15, 27, 28]) in brown; and previously published samples dating to the post-Black Death period (15-18th centuries, [24, 27]) in blue.

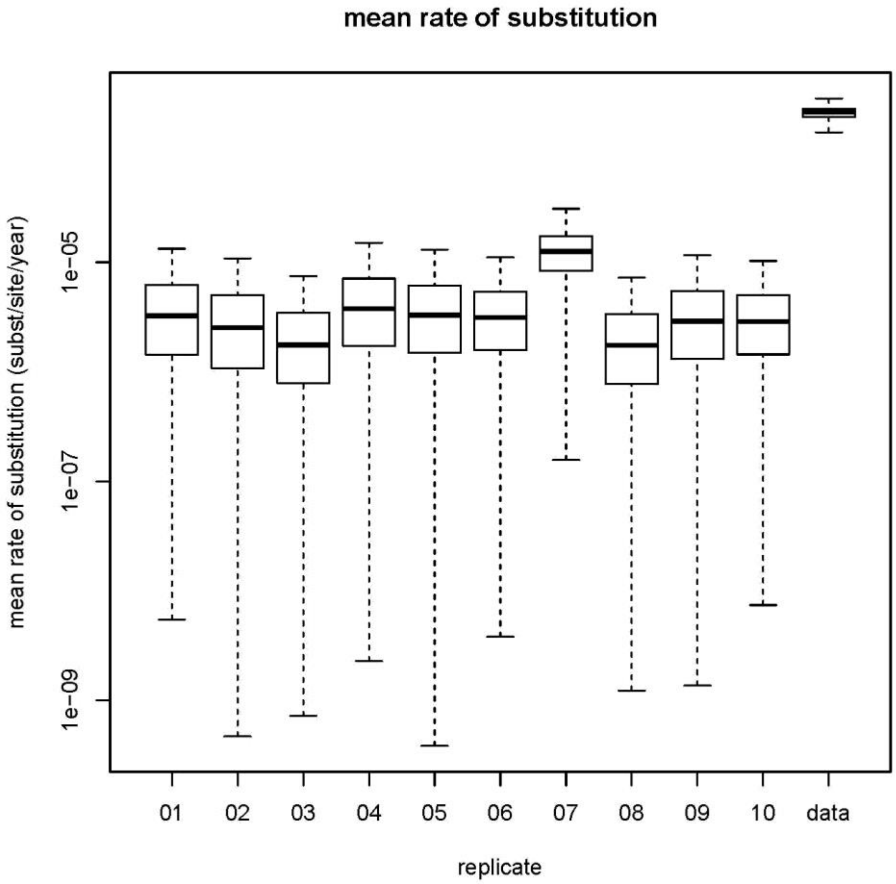

Supplementary Figure 7. Results of Date-Randomization Test for the plague BEAST dataset. Estimates for ten replicates with tip dates randomized among the samples (1-10) and for the original data ("data"). The lack of overlap between the original estimate values and the estimates for the replicates indicate that the dataset represents a measurably evolving population, i.e. the temporal signal in the dataset supports the applicability of tip dating analysis.

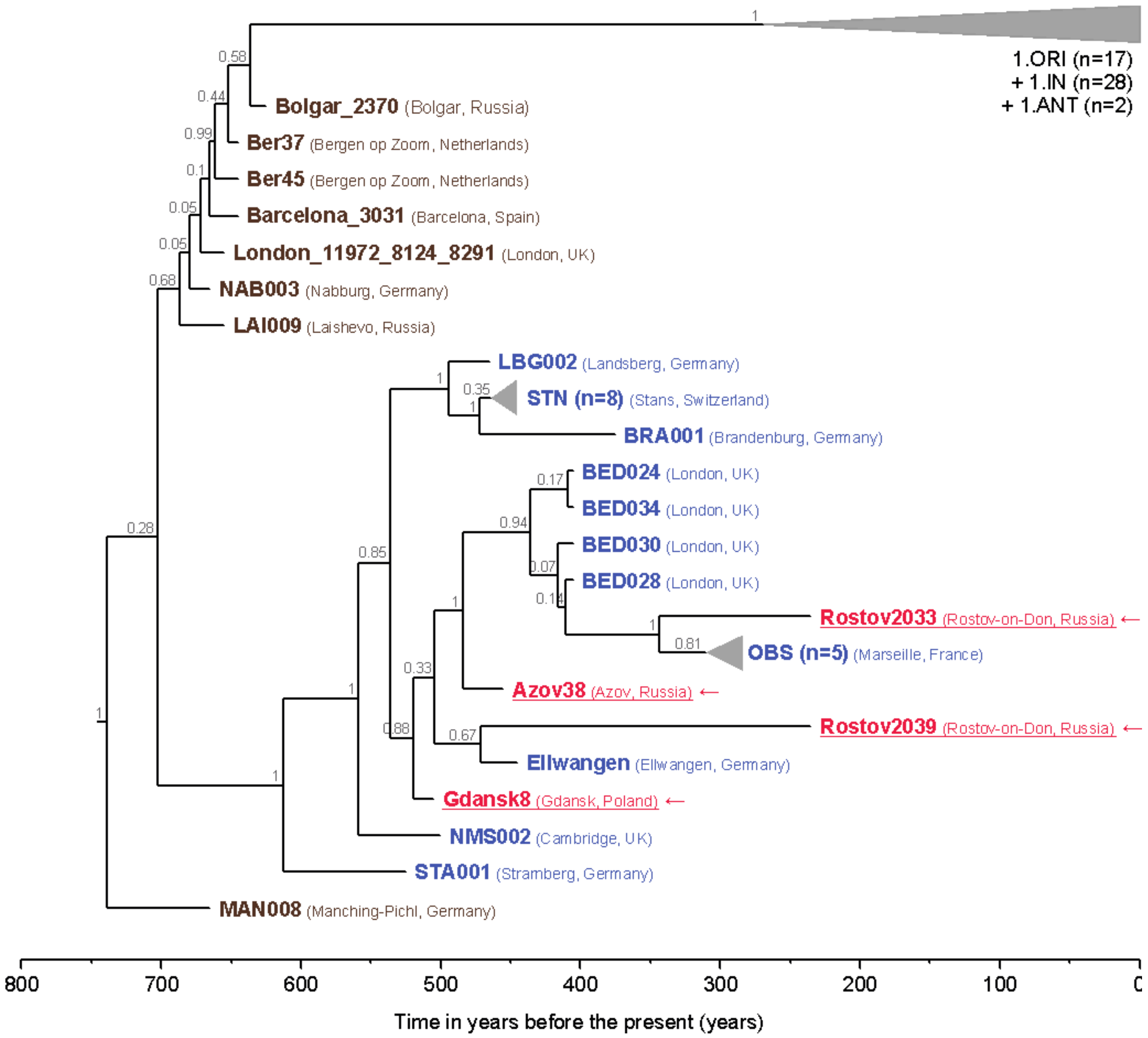

Supplementary Figure 8. Bayesian dated Maximum Clade Credibility tree for Branch 1 generated using BEAST [40] with tip dating molecular clock calibration. The newly studied *Y. pestis* genomes (15-18th centuries) are in red and marked by arrows. The previously published samples dating closer to the Black Death period (13-14th centuries) are marked in brown. The previously published samples dated to the post-Black Death period (15-18th centuries) are marked in blue. The modern *Y. pestis* strains are collapsed to improve the tree visibility. The number of samples inside the collapsed branches are indicated in brackets. For detailed information about the strains included in the analysis, see Supplementary Table 2 and Supplementary Figure 3. Node labels are Bayesian posterior probabilities. Scale shown in years before the present where the present is the date of the youngest sample, i.e. year 2005.

Supplementary references

1 Bell, W. G. 1995 The Great Plague of London. *Random House UK Ltd*.

2 Kislichkina, A. A., Bogun, A. G., Kadnikova, L. A., Maiskaya, N. V., Platonov, M. E., Anisimov, N. V., Galkina, E. V., Dentovskaya, S. V., Anisimov, A. P. 2015 Nineteen Whole-Genome Assemblies of *Yersinia pestis* subsp. microtus, Including Representatives of Biovars caucasica, talassica, hissarica, altaica, xilingolensis, and ulegeica. *Genome Announcements*. **3**. (10.1128/genomeA.01342-15)

3 Russia. 1839 Polnoe sobranie zakonov Rossiiskoi Imperii = Complete collection of the laws of the Russian empire. *St. Petersburg.*

4 Szyszka, M. 2017 Ossuaria w kontekście badań archeologicznych Kępy Dominikańskiej. In: *Nowożytne ossuaria z klasztoru dominikańskiego w Gdańsku. Wyniki badań interdyscyplinarnych. Fontes Commentationesque ad Res Gestas Gedani et Pomeraniae 6.* (ed.^eds. A. Pudło, M. Henneberg), pp. 47-68. Gdańsk: Muzeum Archeologiczne w Gdańsku.

5 Trawicka, E. 2017 Zabytki metalowe z ossuariów odkrytych na Kępie Dominikańskiej w Gdańsku. In: *Nowożytne ossuaria z klasztoru dominikańskiego w Gdańsku. Wyniki badań interdyscyplinarnych. Fontes Commentationesque ad Res Gestas Gedani et Pomeraniae 6*. (ed.^eds. A. Pudło, M. Henneberg), pp. 69-86. Gdańsk: Muzeum Archeologiczne w Gdańsku.

6 Morozova, I., Cieślik, A., Rühli, F. 2017 Genetic analysis of plague in Gdańsk ossuaries (15 - 17th centuries): first findings. In: *Nowożytne ossuaria z klasztoru dominikańskiego w Gdańsku. Wyniki badań interdyscyplinarnych. Fontes Commentationesque ad Res Gestas Gedani et Pomeraniae 6*. (ed.^eds. A. Pudło, M. Henneberg), pp. 255-260. Gdańsk: Muzeum Archeologiczne w Gdańsku.

7 Cooper, A., Poinar, H. N. 2000 Ancient DNA: do it right or not at all. *Science*. **289**, 1139. (10.1126/science.289.5482.1139b)

8 Knapp, M., Lalueza-Fox, C., Hofreiter, M. 2015 Re-inventing ancient human DNA. *Investig Genet*. **6**, 4. (10.1186/s13323-015-0020-4)

9 Rogaev, E. I., Grigorenko, A. P., Moliaka, Y. K., Faskhutdinova, G., Goltsov, A., Lahti, A., Hildebrandt, C., Kittler, E. L., Morozova, I. 2009 Genomic identification in the historical case of the Nicholas II royal family. *Proc Natl Acad Sci U S A*. **106**, 5258-5263. (10.1073/pnas.0811190106)

10 Schuenemann, V. J., Bos, K., DeWitte, S., Schmedes, S., Jamieson, J., Mittnik, A., Forrest, S., Coombes, B. K., Wood, J. W., Earn, D. J. D., *et al.* 2011 Targeted enrichment of ancient pathogens yielding the pPCP1 plasmid of *Yersinia pestis* from victims of the Black Death. *Proceedings of the National Academy of Sciences*. **108**, E746. (10.1073/pnas.1105107108)

11 Harbeck, M., Seifert, L., Hänsch, S., Wagner, D. M., Birdsell, D., Parise, K. L., Wiechmann, I., Grupe, G., Thomas, A., Keim, P., *et al.* 2013 *Yersinia pestis* DNA from Skeletal Remains from the

6th Century AD Reveals Insights into Justinianic Plague. *PLOS Pathogens*. **9**, e1003349. (10.1371/journal.ppat.1003349)

12 Chakravorty, S., Helb, D., Burday, M., Connell, N., Alland, D. 2007 A detailed analysis of 16S ribosomal RNA gene segments for the diagnosis of pathogenic bacteria. *J Microbiol Methods*. **69**, 330-339. (10.1016/j.mimet.2007.02.005)

13 Meyer, M., Kircher, M. 2010 Illumina Sequencing Library Preparation for Highly Multiplexed Target Capture and Sequencing. *Cold Spring Harbor protocols*. **2010**, pdb.prot5448. (10.1101/pdb.prot5448)

14 Kircher, M., Sawyer, S., Meyer, M. 2012 Double indexing overcome inaccuracies in multiplex sequencing on the Illumina platform. *Nucleic acids research*. **40**, e3. (10.1093/nar/gkr771)

15 Bos, K. I., Schuenemann, V. J., Golding, G. B., Burbano, H. A., Waglechner, N., Coombes, B. K., McPhee, J. B., DeWitte, S. N., Meyer, M., Schmedes, S., *et al.* 2011 A draft genome of *Yersinia pestis* from victims of the Black Death. *Nature*. **478**, 506-510. (10.1038/nature10549)

16 Burbano, H. A., Hodges, E., Green, R. E., Briggs, A. W., Krause, J., Meyer, M., Good, J. M., Maricic, T., Johnson, P. L., Xuan, Z., *et al.* 2010 Targeted investigation of the Neandertal genome by array-based sequence capture. *Science*. **328**, 723-725. (10.1126/science.1188046)

17 Peltzer, A., Jager, G., Herbig, A., Seitz, A., Kniep, C., Krause, J., Nieselt, K. 2016 EAGER: efficient ancient genome reconstruction. *Genome Biol*. **17**, 60. (10.1186/s13059-016-0918-z)

18 Andrews, S. 2010 FastQC: a quality control tool for high throughput sequence data. *Babraham Bioinformatics*.

19 Schubert, M., Lindgreen, S., Orlando, L. 2016 AdapterRemoval v2: rapid adapter trimming, identification, and read merging. *BMC Research Notes*. **9**, 88. (10.1186/s13104-016-1900-2)

20 Neukamm, J., Peltzer, A. 2018 Integrative-Transcriptomics/DamageProfiler v0.3.12. (10.5281/ZENODO.1288880)

21 DePristo, M. A., Banks, E., Poplin, R., Garimella, K. V., Maguire, J. R., Hartl, C., Philippakis, A. A., del Angel, G., Rivas, M. A., Hanna, M., *et al.* 2011 A framework for variation discovery and genotyping using next-generation DNA sequencing data. *Nat Genet*. **43**, 491-498. (10.1038/ng.806)

22 Van der Auwera, G. A., Carneiro, M. O., Hartl, C., Poplin, R., Del Angel, G., Levy-Moonshine, A., Jordan, T., Shakir, K., Roazen, D., Thibault, J., *et al.* 2013 From FastQ data to high confidence variant calls: the Genome Analysis Toolkit best practices pipeline. *Curr Protoc Bioinformatics*. **43**, 11 10 11-11 10 33. (10.1002/0471250953.bi1110s43)

23 Li, H., Durbin R. 2010 Fast and accurate long-read alignment with Burrows–Wheeler transform. *Bioinformatics*. **26.5**, 589-595.

24 Bos, K. I., Herbig, A., Sahl, J., Waglechner, N., Fourment, M., Forrest, S. A., Klunk, J., Schuenemann, V. J., Poinar, D., Kuch, M., *et al.* 2016 Eighteenth century *Yersinia pestis* genomes reveal the long-term persistence of an historical plague focus. *Elife*. **5**, e12994. (10.7554/eLife.12994)

25 Cui, Y., Yu, C., Yan, Y., Li, D., Li, Y., Jombart, T., Weinert, L. A., Wang, Z., Guo, Z., Xu, L., *et al.* 2013 Historical variations in mutation rate in an epidemic pathogen, *Yersinia pestis*. *Proceedings of the National Academy of Sciences*. **110**, 577-582. (10.1073/pnas.1205750110)

26 Rasmussen, S., Allentoft, M. E., Nielsen, K., Orlando, L., Sikora, M., Sjogren, K. G., Pedersen, A. G., Schubert, M., Van Dam, A., Kapel, C. M., *et al.* 2015 Early divergent strains of *Yersinia pestis* in Eurasia 5,000 years ago. *Cell*. **163**, 571-582. (10.1016/j.cell.2015.10.009)

27 Spyrou, M. A., Keller, M., Tukhbatova, R. I., Scheib, C. L., Nelson, E. A., Andrades Valtueña, A., Neumann, G. U., Walker, D., Alterauge, A., Carty, N., *et al.* 2019 Phylogeography of the second plague pandemic revealed through analysis of historical *Yersinia pestis* genomes. *Nature Communications*. **10**, 4470. (10.1038/s41467-019-12154-0)

28 Spyrou, M. A., Tukhbatova, R. I., Feldman, M., Drath, J., Kacki, S., Beltran de Heredia, J., Arnold, S., Sitdikov, A. G., Castex, D., Wahl, J., *et al.* 2016 Historical *Y. pestis* Genomes Reveal the European Black Death as the Source of Ancient and Modern Plague Pandemics. *Cell Host Microbe*. **19**, 874-881. (10.1016/j.chom.2016.05.012)

29 Namouchi, A., Guellil, M., Kersten, O., Hänsch, S., Ottoni, C., Schmid, B. V., Pacciani, E., Quaglia, L., Vermunt, M., Bauer, E. L., *et al.* 2018 Integrative approach using *Yersinia pestis* genomes to revisit the historical landscape of plague during the Medieval Period. *Proceedings of the National Academy of Sciences*. **115**, E11790. (10.1073/pnas.1812865115)

30 Feldman, M., Harbeck, M., Keller, M., Spyrou, M. A., Rott, A., Trautmann, B., Scholz, H. C., Paffgen, B., Peters, J., McCormick, M., *et al.* 2016 A High-Coverage *Yersinia pestis* Genome from a Sixth-Century Justinianic Plague Victim. *Molecular Biology and Evolution*. **33**, 2911-2923. (10.1093/molbev/msw170)

31 Eroshenko, G. A., Nosov, N. Y., Krasnov, Y. M., Oglodin, Y. G., Kukleva, L. M., Guseva, N. P., Kuznetsov, A. A., Abdikarimov, S. T., Dzhaparova, A. K., Kutyrev, V. V. 2017 *Yersinia pestis* strains of ancient phylogenetic branch 0.ANT are widely spread in the high-mountain plague foci of Kyrgyzstan. *PLoS One*. **12**, e0187230. (10.1371/journal.pone.0187230)

32 Kutyrev, V. V., Eroshenko, G. A., Motin, V. L., Nosov, N. Y., Krasnov, J. M., Kukleva, L. M., Nikiforov, K. A., Al'khova, Z. V., Oglodin, E. G., Guseva, N. P. 2018 Phylogeny and Classification of *Yersinia pestis* Through the Lens of Strains From the Plague Foci of Commonwealth of Independent States. *Front Microbiol*. **9**, 1106. (10.3389/fmicb.2018.01106)

33 Zhgenti, E., Johnson, S. L., Davenport, K. W., Chanturia, G., Daligault, H. E., Chain, P. S., Nikolich, M. P. 2015 Genome Assemblies for 11 *Yersinia pestis* Strains Isolated in the Caucasus Region. *Genome Announcements*. **3**. (10.1128/genomeA.01030-15)

34 Spyrou, M. A., Tukhbatova, R. I., Wang, C. C., Valtuena, A. A., Lankapalli, A. K., Kondrashin, V. V., Tsybin, V. A., Khokhlov, A., Kuhnert, D., Herbig, A., *et al.* 2018 Analysis of 3800-year-old *Yersinia pestis* genomes suggests Bronze Age origin for bubonic plague. *Nature Communications*. **9**, 2234. (10.1038/s41467-018-04550-9)

35 Keller, M., Spyrou, M. A., Scheib, C. L., Neumann, G. U., Kropelin, A., Haas-Gebhard, B., Paffgen, B., Haberstroh, J., Ribera, I. L. A., Raynaud, C., *et al.* 2019 Ancient *Yersinia pestis* genomes from across Western Europe reveal early diversification during the First Pandemic (541-750). *Proceedings of the National Academy of Sciences*. **116**, 12363-12372. (10.1073/pnas.1820447116)

36 Vagene, A. J., Herbig, A., Campana, M. G., Robles Garcia, N. M., Warinner, C., Sabin, S., Spyrou, M. A., Andrades Valtuena, A., Huson, D., Tuross, N., *et al.* 2018 Salmonella enterica genomes from victims of a major sixteenth-century epidemic in Mexico. *Nature Ecology and Evolution*. **2**, 520-528. (10.1038/s41559-017-0446-6)

37 National Center for Biotechnology Information (NCBI)[Internet]. Bethesda (MD): National Library of Medicine (US), National Center for Biotechnology Information; [1988] – [cited 2018 May]. Available from: https://www.ncbi.nlm.nih.gov/

38 Huson, D. H., Beier, S., Flade, I., Górska, A., El-Hadidi, M., Mitra, S., Ruscheweyh, H.-J., Tappu, R. 2016 MEGAN Community Edition - Interactive Exploration and Analysis of Large-Scale Microbiome Sequencing Data. *PLOS Computational Biology*. **12**, e1004957. (10.1371/journal.pcbi.1004957)

39 Stamatakis, A. 2014 RAxML version 8: a tool for phylogenetic analysis and post-analysis of large phylogenies. *Bioinformatics*. **30**, 1312-1313. (10.1093/bioinformatics/btu033)

40 Suchard, M. A., Lemey, P., Baele, G., Ayres, D. L., Drummond, A. J., Rambaut, A. 2018 Bayesian phylogenetic and phylodynamic data integration using BEAST 1.10. *Virus Evolution*. **4**, (10.1093/ve/vey016)

41 Keane, T. M., Creevey, C. J., Pentony, M. M., Naughton, T. J., McLnerney, J. O. 2006 Assessment of methods for amino acid matrix selection and their use on empirical data shows that ad hoc assumptions for choice of matrix are not justified. *BMC Evolutionary Biology*. **6**, 29. (10.1186/1471-2148-6-29)

42 Rambaut, A., Drummond, A. J., Xie, D., Baele, G., Suchard, M. A. 2018 Posterior Summarization in Bayesian Phylogenetics Using Tracer 1.7. *Syst Biol*. **67**, 901-904. (10.1093/sysbio/syy032)

43 Ramsden, C., Holmes, E. C., Charleston, M. A. 2009 Hantavirus evolution in relation to its rodent and insectivore hosts: no evidence for codivergence. *Mol Biol Evol*. **26**, 143-153. (10.1093/molbev/msn234)

44 Duchene, S., Lemey, P., Stadler, T., Ho, S. Y. W., Duchene, D. A., Dhanasekaran, V., Baele, G. 2019 Bayesian Evaluation of Temporal Signal in Measurably Evolving Populations. *bioRxiv*. 810697. (10.1101/810697)

45 Rambaut, A., Lam, T. T., Carvalho, L. M., Pybus. O. G. 2009 Exploring the temporal structure of heterochronous sequences using TempEst (formerly Path-O-Gen). *Virus Evolution,* Volume **2**, Issue 1, vew007. (10.1093/ve/vew007)

46 Krzywinski, M., Schein, J., Birol, I., Connors, J., Gascoyne, R., Horsman, D., Jones, S. J., Marra, M. A. 2009 Circos: an information aesthetic for comparative genomics. *Genome Res*. **19**, 1639-1645. (10.1101/gr.092759.109)

47 Cingolani, P., Platts, A., Wang le, L., Coon, M., Nguyen, T., Wang, L., Land, S. J., Lu, X., Ruden, D. M. 2012 A program for annotating and predicting the effects of single nucleotide polymorphisms, SnpEff: SNPs in the genome of Drosophila melanogaster strain w1118; iso-2; iso-3. *Fly (Austin)*. **6**, 80-92. (10.4161/fly.19695)

48 Chain, P. S. G., Carniel, E., Larimer, F. W., Lamerdin, J., Stoutland, P. O., Regala, W. M., Georgescu, A. M., Vergez, L. M., Land, M. L., Motin, V. L., *et al.* 2004 Insights into the evolution of *Yersinia pestis* through whole-genome comparison with Yersinia pseudotuberculosis. *Proceedings of the National Academy of Sciences*. **101**, 13826. (10.1073/pnas.0404012101)

Supplementary Table 1. Archaeological information about the studied samples from Eastern Europe

Sample laboratory ID	Archaeological ID	Archaeological date	Sex	Age	Sample	Abbreviation in the text
**Rostov-on-Don, Russia**						
1128	MG-98.sit 1	18th century	M?	25-30	Tooth	
1129	MG-98.gr.1	18th century	M	25-30	Tooth	
1130	MG-98.gr.1 middle	18th century	F	30-35	Tooth	
1131	MG-98.gr.1 northern	18th century	M?	25-30	Tooth	
1132	MG-98.gr.4	18th century	M	25-30	Tooth	
1133	MG-98.gr.5	18th century	M	25-30	Tooth	
1134	MG-98.gr.6	18th century	M?	30-35	Tooth	
1136	MG-98.gr.8	18th century	F	20-25	Tooth	
1137	MG-98.gr.8	18th century	M?	>45	Tooth	
1138	MG-98.gr.10 upper nothern	18th century	F	20-25	Tooth	
1139	MG-98.gr.10 lower central	18th century	M	30-35	Tooth	
1140	MG-98.gr.10 lower	18th century	F	18-20	Tooth	
1141	MG-98.10 upper southern	18th century	F	20-25	Tooth	
1142	MG-98.gr.12	18th century	F?	30-35	Tooth	
1145	MG-98.	18th century	F	25-30	Tooth	
1354	MG-2000.gr.1.№7	18th century	M	20-25	Tooth	
1625	MG -01.gr.5 upper southern (1st tier)	18th century	M	30-35	Tooth	
1627	MG -01.gr.5 lower tier (with cross)	18th century	F	35-40	Tooth	
1628	MG -01.gr.5 lower tier	18th century	M	25-30	Tooth	
1629	MG -01.gr.5 lower tier southward coffin	18th century	F	30-35	Tooth	
1631	MG -01.gr.6 norhtern middle southern	18th century	F	25-30	Tooth	
1632	MG -01.gr.6 above the coffin	18th century	M	25-30	Tooth	
1633	MG -01.gr.6 above the coffin	18th century	M	35-40	Tooth	
1635	MG -01.gr.9 northern	18th century	F	35-40	Tooth	
1636	MG -01.gr.13 upper southern	18th century	M	35-40	Tooth	
1639	MG -01.gr.16 upper southern	18th century	M	30-35	Tooth	
1640	MG -01.gr.16 lower southern	18th century	F	25-30	Tooth	

Supplementary Table 1 (continued)

**1639a**	**MG -01.gr.16 lower southern**	**18th century**	**F?**	**35-40**	**Tooth**	**Rostov1639**
1641	MG -01.gr.19 lower southern	18th century	M	25-30	Tooth	
1642	MG -01.gr.19 upper southern	18th century	F	25-30	Tooth	
1643	MG -01.gr.19 northern	18th century	M	25-30	Tooth	
1646	MG -01.gr.23 northern lower tier	18th century	M	30-35	Tooth	
1651	MG -01.gr.28	18th century	F	20-25	Tooth	
1654	MG -01.gr.31	18th century	F	25-30	Tooth	
2031	ROI-04-MG.gr.1	1762-1773	?	25-35	Tooth	
**2033**	**ROI-04-MG.gr.1**	**1762-1773**	**F**	**17-25**	**Tooth**	**Rostov2033**
2034	ROI-04-MG.gr.2	1762-1773	M	17-25	Tooth	
**2039**	**ROI-04-MG.gr.8 lower southern**	**1762-1773**	**M**	**30-35**	**Tooth**	**Rostov2039**
2040	ROI-04-MG.gr.8 lower northern	1762-1773	F?	20-25	Tooth	
**Azov, Russia**						
N37	Azov-2005 Lermontova N37 gr. 25	15th-17th century	M	>35	Petrous bone	
N14	Azov-2008 Chapaeva 14 gr.11	16th-18th century	M	35-45	Tooth	
N27	Azov-2012 Lermontova 27 sect. 1 gr.7	15th-17th century	F	>30	Petrous bone	
**N38**	**Azov-2012 Kalinina 38 gr.5**	**15th-17th century**	**M**	**25-35**	**Tooth**	**Azov38**
**Gdańsk, Poland**						
O5	255/05/08 Oss. 3009 w. 3010 k. 272/2014 No.1	15th-16th centuries	?	20-30	Tooth	
O6	255/05/08 Oss. 3009 w. 3010 k. 287/2014 No.2	15th-16th centuries	?	35-45	Tooth	
O7	255/05/08 Oss. 3009 w. 3010 k. 440/2014 No.3	15th-16th centuries	M	35-50	Tooth	
O21	255/05/08 Oss. 3009 w. 3010 k. 317/2014 No.5	15th-16th centuries	?	25-30	Tooth	
O19	255/05/08 Oss. 3009 w. 3010 k. 317/2014 No.6	15th-16th centuries	F	15-18	Tooth	
O22	255/05/08 Oss. 3009 w. 3010 k. 274/2014 No.7	15th-16th centuries	M	30-40	Tooth	
O24	255/05/08 Oss. 3009 w. 3010 k. 344/2014 No.8	15th-16th centuries	M	20-30	Tooth	
O23	255/05/08 Oss. 3009 w. 3010 k. 318/2014 No.9	15th-16th centuries	?	30-40	Tooth	
O9	255/05/08 Oss. 3009 w. 3010 k. 271/2014 No.10	15th-16th centuries	?	30-35	Tooth	
**O8**	**255/05/08 Oss. 3009 w. 3010 k. 243/2014 No.11**	**1425-1469***	**M**	**25-30**	**Tooth**	**Gdansk8**
O20	255/05/08 Oss. 3009 w. 3010 k. 243/2014 No.12	15th-16th centuries	M	30-35	Tooth	
O18	255/05/08 Oss. 3009 w. 3010 k. 349/2014 No.13	15th-16th centuries	F	30-40	Tooth	

Supplementary Table 1 (continued)

**Rat**	**Oss. 3009 w. 2010**	**15th-16th centuries**	**NA**	**NA**	**Skull**	**Rat**
O4	255/05/08 Oss. 2006 w. 2007 k. 91/2014 No.1	18th century	M	30-40	Tooth	
O11	255/05/08 Oss. 2006 w. 2007 k. 91/2014 No.2	18th century	M	30-40	Tooth	
O29	255/05/08 Oss. 2006 w. 2007 k. 91/2014 No.3	18th century	M	30-40	Tooth	
O25	255/05/08 Oss. 2006 w. 2007 k. 92/2014 No.4	18th century	M	30-35	Tooth	
O3	255/05/08 Oss. 2006 w. 2007 k. 92/2014 No.5	18th century	M	30-40	Tooth	
O2	255/05/08 Oss. 2006 w. 2007 k. 92/2014 No.6	18th century	F	18-20	Tooth	
O10	255/05/08 Oss. 2006 w. 2007 k. 92/2014 No.7	18th century	M	40-50	Tooth	
O28	255/05/08 Oss. 2006 w. 2007 k. 92/2014 No.8	18th century	F	20-30	Tooth	
O26	255/05/08 Oss. 2006 w. 2007 k. 92/2014 No.9	18th century	M	30-40	Tooth	
O1	255/05/08 Oss. 2006 w. 2007 k. 92/2014 No.10	18th century	F	16-18	Tooth	
O31	255/05/08 Oss. 2046 w. 2047 k. 246 No.1	18th century	?	30-40	Tooth	
O30	255/05/08 Oss. 2046 w. 2047 k. 246 No.2	18th century	?	50+	Tooth	
O33	255/05/08 Oss. 2046 w. 2047 k. 304/2014 No.3	18th century	M?	45-55	Tooth	
O12	255/05/08 Oss. 2046 w. 2047 k. 304/2014 No.4	18th century	F	45-55	Tooth	
O27	255/05/08 Oss. 2046 w. 2047 k. 246 No.5	18th century	M	20-30	Tooth	
O34	255/05/08 Oss. 2046 w. 2047 k. 246 No.6	18th century	M?	45-55	Tooth	
O32	255/05/08 Oss. 2046 w. 2047 k. 246 No.7	18th century	M	35-45	Tooth	
O13	255/05/08 Oss. 2046 w. 2047 k. 246 No.8	18th century	M	30-35	Tooth	
O15	255/05/08 Oss. 2046 w. 2047 k. 246 No.9	18th century	F	16-18	Tooth	
O16	255/05/08 Oss. 2046 w. 2047 k. 246 No.11	18th century	F	35-45	Tooth	
O17	255/05/08 Oss. 2046 w. 2047 k. 246 No.12	18th century	M	30-40	Tooth	
O14	255/05/08 Oss. 2046 w. 2047 k. 246 No.14	18th century	M?	30-40	Tooth	
O35	255/05/08 Oss. 2046 w. 2047 k. 182 No.15	18th century	M	30-35	Tooth	

Note. Plague-positive samples are in bold

*The dates are based on ^{14}C dating. All other dates are based on archaeological information

Supplementary Table 2. Published data used in phylogenetic analysis

Sample	Used for	Publication (or NCBI accession)	Dating	Isolate	Geographic location
Azov38	BEAST / ML tree	This study	1400-1700	Ancient plague	Azov, Russian Federation
Gdansk8	BEAST / ML tree	This study	1400-1600	Ancient plague	Gdańsk, Poland
Rostov2033	BEAST / ML tree	This study	1762-1773	Ancient plague	Rostov-on-Don, Russian Federation
Rostov2039	BEAST / ML tree	This study	1762-1773	Ancient plague	Rostov-on-Don, Russian Federation
1.ANT1_Antiqua	BEAST / ML tree	NC_008150	1965	1.ANT1	Congo
1.ANT1_UG05-0454	BEAST / ML tree	NZ_AAYR01000000	2004	1ANT1	Uganda
1.IN1a_CMCC11001	BEAST / ML tree	Cui et al., 2013	1954	1.IN1a	Qinghai, China
1.IN1b_780441	BEAST / ML tree	Cui et al., 2013	1978	1.IN1b	Qinghai, China
1.IN1c_K21985002	BEAST / ML tree	Cui et al., 2013	1985	1.IN1c	Xinjiang, China
1.IN2a_CMCC640047	BEAST / ML tree	Cui et al., 2013	1964	1.IN2a	Qinghai, China
1.IN2b_30017	BEAST / ML tree	Cui et al., 2013	1976	1.IN2b	Tibet, China
1.IN2c_CMCC31004	BEAST / ML tree	Cui et al., 2013	1990	1.IN2c	Tibet, China
1.IN2d_C1975003	BEAST / ML tree	Cui et al., 2013	1975	1.IN2d	Qinghai, China
1.IN2e_C1989001	BEAST / ML tree	Cui et al., 2013	1989	1.IN2e	Qinghai, China
1.IN2f_710317	BEAST / ML tree	Cui et al., 2013	1971	1.IN2f	Qinghai, China
1.IN2g_CMCC05013	BEAST / ML tree	Cui et al., 2013	1988	1.IN2g	Qinghai, China
1.IN2h_5	BEAST / ML tree	Cui et al., 2013	2004	1.IN2h	Qinghai, China
1.IN2i_CMCC10012	BEAST / ML tree	Cui et al., 2013	1964	1.IN2i	Qinghai, China
1.IN2j_CMCC27002	BEAST / ML tree	Cui et al., 2013	1991	1.IN2j	Qinghai, China
1.IN2k_970754	BEAST / ML tree	Cui et al., 2013	1997	1.IN2k	Qinghai, China
1.IN2l_D1991004	BEAST / ML tree	Cui et al., 2013	1991	1.IN2l	Qinghai, China
1.IN2m_D1964002b	BEAST / ML tree	Cui et al., 2013	1964	1.IN2m	Qinghai, China
1.IN2n_CMCC02041	BEAST / ML tree	Cui et al., 2013	1965	1.IN2n	Qinghai, China
1.IN2o_CMCC03001	BEAST / ML tree	Cui et al., 2013	1954	1.IN2o	Qinghai, China
1.IN2p_D1982001	BEAST / ML tree	Cui et al., 2013	1982	1.IN2p	Gansu, China
1.IN2q_D1964001	BEAST / ML tree	Cui et al., 2013	1964	1.IN2q	Qinghai, China
1.IN3a_F1954001	BEAST / ML tree	Cui et al., 2013	1954	1.IN3a	Yunnan, China
1.IN3b_E1979001	BEAST / ML tree	Cui et al., 2013	1979	1.IN3b	Yunnan, China
1.IN3c_CMCC84038b	BEAST / ML tree	Cui et al., 2013	1982	1.IN3c	Yunnan, China

Supplementary Table 2 (continued)

1.IN3d_YN1683	BEAST / ML tree	Cui et al., 2013	1977	1.IN3d	Yunnan, China
1.IN3e_YN472	BEAST / ML tree	Cui et al., 2013	1957	1.IN3e	Yunnan, China
1.IN3f_YN1065	BEAST / ML tree	Cui et al., 2013	1954	1.IN3f	Yunnan, China
1.IN3g_E1977001	BEAST / ML tree	Cui et al., 2013	1977	1.IN3g	Yunnan, China
1.IN3h_CMCC84033	BEAST / ML tree	Cui et al., 2013	1979	1.IN3h	Yunnan, China
1.IN3i_CMCC84046	BEAST / ML tree	Cui et al., 2013	1984	1.IN3i	Yunnan, China
1.ORI1a_CMCC114001	BEAST / ML tree	Cui et al., 2013	1952	1.ORI1a	Fujian, China
1.ORI1b_India195	BEAST / ML tree	NZ_ACNR00000000	1898	1.ORI1b	India
1.ORI1c_F1946001	BEAST / ML tree	Cui et al., 2013	1946	1.ORI1c	Fujian, China
1.ORI1d_CA88	BEAST / ML tree	NZ_ABCD00000000	1988	1.ORI1d	California, USA
1.ORI1e_CO92	BEAST / ML tree	NC_003143	1992	1.ORI1d	Colorado, USA
1.ORI2a_YN2179	BEAST / ML tree	Cui et al., 2013	1995	1.ORI2a	Myanmar
1.ORI2b_CMCCK110001	BEAST / ML tree	Cui et al., 2013	1991	1.ORI2b	Yunnan, China
1.ORI2c_YN2551	BEAST / ML tree	Cui et al., 2013	2002	1.ORI2c	Yunnan, China
1.ORI2d_YN2588	BEAST / ML tree	Cui et al., 2013	2000	1.ORI2d	Guangxi, China
1.ORI2e_F1991016	BEAST / ML tree	NZ_ABAT00000000	1991	1.ORI2e	Yunnan, China
1.ORI2f_CMCC87001	BEAST / ML tree	Cui et al., 2013	1982	1.ORI2f	Yunnan, China
1.ORI2g_F1984001	BEAST / ML tree	Cui et al., 2013	1984	1.ORI2g	Yunnan, China
1.ORI2h_YN663	BEAST / ML tree	Cui et al., 2013	1982	1.ORI2h	Yunnan, China
1.ORI2i_CMCCK100001	BEAST / ML tree	Cui et al., 2013	1984	1.ORI2i	Yunnan, China
1.ORI3a_EV76	BEAST / ML tree	Cui et al., 2013	1922	1.ORI3a	Madagascar
1.ORI3b_MG05-1020	BEAST / ML tree	NZ_AAYS00000000	2005	1.ORI3b	Madagascar
1.ORI3c_IP275	BEAST / ML tree	AAOS02000088	1995	1.ORI3c	Madagascar
Barcelona_3031	BEAST / ML tree	Spyrou et al., 2016	1300-1420	Ancient plague	Barcelona, Spain
BED024	BEAST / ML tree	Spyrou et al., 2019	1560-1635	Ancient plague	London, Great Britain
BED028	BEAST / ML tree	Spyrou et al., 2019	1560-1635	Ancient plague	London, Great Britain
BED030	BEAST / ML tree	Spyrou et al., 2019	1560-1635	Ancient plague	London, Great Britain
BED034	BEAST / ML tree	Spyrou et al., 2019	1560-1635	Ancient plague	London, Great Britain
Ber37	BEAST / ML tree	Namouchi et al., 2018	1358-1360	Ancient plague	Bergen op Zoom, Netherlands
Ber45	BEAST / ML tree	Namouchi et al., 2018	1358-1360	Ancient plague	Bergen op Zoom, Netherlands

Supplementary Table 2 (continued)

Bolgar_2370	BEAST / ML tree	Spyrou et al., 2016	1362-1400	Ancient plague	Bolgar, Russian Federation
BRA001	BEAST / ML tree	Spyrou et al., 2019	1618-1648	Ancient plague	Brandenburg, Germany
Ellwangen	BEAST / ML tree	Spyrou et al., 2016	1485-1627	Ancient plague	Ellwangen, Germany
LAI009	BEAST / ML tree	Spyrou et al., 2019	1300-1400	Ancient plague	Laishevo, Russian Federation
LBG002	BEAST / ML tree	Spyrou et al., 2019	1455-1634	Ancient plague	Landsberg, Germany
London_11972_8124_8291	BEAST / ML tree	Bos et al., 2011	1348-1350	Ancient plague	London, Great Britain
MAN008	BEAST / ML tree	Spyrou et al., 2019	1283-1390	Ancient plague	Manching, Germany
NAB003	BEAST / ML tree	Spyrou et al., 2019	1292-1392	Ancient plague	Nabburg, Germany
NMS002.A	BEAST / ML tree	Spyrou et al., 2019	1475-1536	Ancient plague	Cambridge, Great Britain
OBS107	BEAST / ML tree	Bos et al., 2016	1722	Ancient plague	Marseille, France
OBS110	BEAST / ML tree	Bos et al., 2016	1722	Ancient plague	Marseille, France
OBS116	BEAST / ML tree	Bos et al., 2016	1722	Ancient plague	Marseille, France
OBS124	BEAST / ML tree	Bos et al., 2016	1722	Ancient plague	Marseille, France
OBS137	BEAST / ML tree	Bos et al., 2016	1722	Ancient plague	Marseille, France
STA001	BEAST / ML tree	Spyrou et al., 2019	1433-1523	Ancient plague	Strarnberg, Germany
STN002	BEAST / ML tree	Spyrou et al., 2019	1485-1635	Ancient plague	Stans, Switzerland
STN007	BEAST / ML tree	Spyrou et al., 2019	1485-1635	Ancient plague	Stans, Switzerland
STN008	BEAST / ML tree	Spyrou et al., 2019	1485-1635	Ancient plague	Stans, Switzerland
STN013	BEAST / ML tree	Spyrou et al., 2019	1485-1635	Ancient plague	Stans, Switzerland
STN014	BEAST / ML tree	Spyrou et al., 2019	1485-1635	Ancient plague	Stans, Switzerland
STN019	BEAST / ML tree	Spyrou et al., 2019	1485-1635	Ancient plague	Stans, Switzerland
STN020	BEAST / ML tree	Spyrou et al., 2019	1485-1635	Ancient plague	Stans, Switzerland
STN021	BEAST / ML tree	Spyrou et al., 2019	1485-1635	Ancient plague	Stans, Switzerland
0.ANT1a_42013	ML tree	Cui et al., 2013		0.ANT1a	Xinjiang, China
0.ANT1b_CMCC49003	ML tree	Cui et al., 2013		0.ANT1b	Xinjiang, China
0.ANT1c_945	ML tree	Cui et al., 2013		0.ANT1c	Xinjiang, China
0.ANT1d_164	ML tree	Cui et al., 2013		0.ANT1d	Xinjiang, China
0.ANT1e_CMCC8211	ML tree	Cui et al., 2013		0.ANT1e	Xinjiang, China
0.ANT1f_42095	ML tree	Cui et al., 2013		0.ANT1f	Xinjiang, China
0.ANT1g_CMCC42007	ML tree	Cui et al., 2013		0.ANT1g	Xinjiang, China

Supplementary Table 2 (continued)

0.ANT1h_CMCC43032	ML tree	Cui et al., 2013		0.ANT1h	Xinjiang, China
0.ANT2a_2330	ML tree	Cui et al., 2013		0.ANT2a	Xinjiang, China
0.ANT2a_B42003004	ML tree	Cui et al., 2013		0.ANT3a	Xinjiang, China
0.ANT3_231	ML tree	Eroshenko et al. 2017		0.ANT3	Aksai high-mountain focus
0.ANT3_790	ML tree	Zhgenti et al., 2015		0.ANT3	Kyrgyzstan
0.ANT3_A-1486	ML tree	Eroshenko et al. 2017		0.ANT3	Aksai high-mountain focus
0.ANT3a_CMCC38001	ML tree	Cui et al., 2013		0.ANT3a	Xinjiang, China
0.ANT3b_A1956001	ML tree	Cui et al., 2013		0.ANT3b	Xinjiang, China
0.ANT3c_42082	ML tree	Cui et al., 2013		0.ANT3c	Xinjiang, China
0.ANT3d_CMCC21106	ML tree	Cui et al., 2013		0.ANT3d	Xinjiang, China
0.ANT3e_42091	ML tree	Cui et al., 2013		0.ANT3e	Xinjiang, China
0.ANT5_262	ML tree	Eroshenko et al. 2017		0.ANT5	Upper-Naryn high-mountain focus
0.ANT5_5M	ML tree	Eroshenko et al. 2018		0.ANT5	Upper-Naryn high-mountain focus
0.ANT5_A-1691	ML tree	Eroshenko et al. 2017		0.ANT5	Sarydzhaz high-mountain focus
0.ANT5_A-1836	ML tree	Eroshenko et al. 2017		0.ANT5	Sarydzhaz high-mountain focus
0.PE2_1412	ML tree	Zhgenti et al., 2015		0.PE2	Georgia
0.PE2_1413	ML tree	Zhgenti et al., 2015		0.PE2	Georgia
0.PE2_14735	ML tree	Zhgenti et al., 2015		0.PE2	Armenia
0.PE2_1522	ML tree	Zhgenti et al., 2015		0.PE2	Armenia
0.PE2_1670	ML tree	Zhgenti et al., 2015		0.PE2	Georgia
0.PE2_3067	ML tree	Zhgenti et al., 2015		0.PE2	Georgia
0.PE2_3544	ML tree	Kutyrev et al., 2018		0.PE2	Leninakan mountain, Armenia
0.PE2_3551	ML tree	Kutyrev et al., 2018		0.PE2	Prisevansky mountain, Armenia
0.PE2_3770	ML tree	Zhgenti et al., 2015		0.PE2	Georgia
0.PE2_835_BPC	ML tree	Kutyrev et al., 2018		0.PE2	Leninakan mountain, Armenia
0.PE2_8787	ML tree	Zhgenti et al., 2015		0.PE2	Georgia
0.PE2_C-741	ML tree	Kutyrev et al., 2018		0.PE2	Russian Federation
0.PE2_KM874	ML tree	Kutyrev et al., 2018		0.PE2	Armenia
0.PE2_M-986	ML tree	Kutyrev et al., 2018		0.PE2	Armenia
0.PE2_SCPM-O-B-6176_C-535	ML tree	PRJNA269675		0.PE2	Dagestan, Russian Federation

Supplementary Table 2 (continued)

0.PE2_SCPM-O-B-6992_C-700	ML tree	PRJNA269675		0.PE2	Dagestan, Russian Federation
0.PE2_SCPM-O-B-6994_C-739	ML tree	PRJNA269675		0.PE2	Dagestan, Russian Federation
0.PE2_SCPM-O-B-7005_C-824	ML tree	PRJNA269675		0.PE2	Dagestan, Russian Federation
0.PE2_SCPM-O-B-7037_C-370	ML tree	PRJNA269675		0.PE2	Dagestan, Russian Federation
0.PE2_SCPM-O-B-7040_C-678	ML tree	PRJNA269675		0.PE2	Dagestan, Russian Federation
0.PE2_SCPM-O-B-7042_C-712	ML tree	PRJNA269675		0.PE2	Dagestan, Russian Federation
0.PE2_SCPM-O-B-7111_C-746	ML tree	PRJNA269675		0.PE2	Dagestan, Russian Federation
0.PE2a_Pestoides_F	ML tree	NC_009381.1		0.PE2a	Former Soviet Union
0.PE2b_G8786	ML tree	Cui et al., 2013		0.PE2b	Georgia
0.PE3a_Angola	ML tree	NC_010159		0.PE3a	Africa
0.PE4a_B1313	ML tree	Kutyrev et al., 2018		0.PE4a	Russian Federation
0.PE4a_I-2751-55	ML tree	Kutyrev et al., 2018		0.PE4a	Russian Federation
0.PE4a_I-2998	ML tree	Kutyrev et al., 2018		0.PE4a	Russian Federation
0.PE4Aa_12	ML tree	Cui et al., 2013		0.PE4Aa	Qinghai, China
0.PE4Ab_9	ML tree	Cui et al., 2013		0.PE4Aa	Qinghai, China
0.PE4Ba_PestoidesA	ML tree	ACNT01000009.1		0.PE4Ab	Former Soviet Union
0.PE4Ca_CMCCN010025	ML tree	Cui et al., 2013		0.PE4Ca	Sichuan, China
0.PE4Cb_M0000002	ML tree	Cui et al., 2013		0.PE4Cb	Qinghai, China
0.PE4Cc_CMCC18019	ML tree	Cui et al., 2013		0.PE4Cc	Qinghai, China
0.PE4Cd_CMCC93014	ML tree	Cui et al., 2013		0.PE4Cd	Inner Mongolia, China
0.PE4Ce_CMCC91090	ML tree	Cui et al., 2013		0.PE4Ce	Inner Mongolia, China
0.PE4Cf_Microtus91001	ML tree	NC_005810		0.PE4Cf	Inner Mongolia, China
0.PE4h_A-1249	ML tree	Eroshenko et al. 2017		0.PE4h	Sogdiyskaya Region, Tajikistan
0.PE4m_I-3086	ML tree	Kutyrev et al., 2018		0.PE4m	Bayan-Khongor aimak, Mongolia
0.PE4t_A-1815	ML tree	Eroshenko et al. 2017		0.PE4t	Talas high-mountain focus
0.PE5_SCPM-O-B-6212_I-2238	ML tree	PRJNA269675		0.PE5	--
0.PE5_SCPM-O-B-6301_I-2231	ML tree	PRJNA269675		0.PE5	--
0.PE5_SCPM-O-DNA-15_I-2236	ML tree	PRJNA269675		0.PE5	--
0.PE5_SCPM-O-DNA-16_I-2422	ML tree	Kislichkina ei al., 2015		0.PE5	--
0.PE7a_CMCC05009	ML tree	Cui et al., 2013		0.PE7a	Qinghai, China

Supplementary Table 2 (continued)

0.PE7b_620024	ML tree	Cui et al., 2013		0.PE7b	Qinghai, China
2.ANT1a_34008	ML tree	Cui et al., 2013		2.ANT1a	Tibet, China
2.ANT1b_34202	ML tree	Cui et al., 2013		2.ANT1b	Tibet, China
2.ANT1c_Nepal516	ML tree	ACNQ01000007		2.ANT1c	Nepal
2.ANT2a_2	ML tree	Cui et al., 2013		2.ANT2a	Qinghai, China
2.ANT2b_351001	ML tree	Cui et al., 2013		2.ANT2b	Tibet, China
2.ANT2c_CMCC347001	ML tree	Cui et al., 2013		2.ANT2c	Tibet, China
2.ANT2d_G1996006	ML tree	Cui et al., 2013		2.ANT2d	Tibet, China
2.ANT2e_G1996010	ML tree	Cui et al., 2013		2.ANT2e	Tibet, China
2.ANT2f_CMCC348002	ML tree	Cui et al., 2013		2.ANT2f	Tibet, China
2.ANT3_KM682_I1996	ML tree	Kutyrev et al., 2018		2.ANT3	Trans-Baikal steppe, Russian Federation
2.ANT3a_CMCC92010	ML tree	Cui et al., 2013		2.ANT3a	Inner Mongolia, China
2.ANT3b_CMCC95001	ML tree	Cui et al., 2013		2.ANT3b	Inner Mongolia, China
2.ANT3c_CMCC96001	ML tree	Cui et al., 2013		2.ANT3c	Inner Mongolia, China
2.ANT3d_CMCC96007	ML tree	Cui et al., 2013		2.ANT3d	Inner Mongolia, China
2.ANT3e_CMCC67001	ML tree	Cui et al., 2013		2.ANT3e	Inner Mongolia, China
2.ANT3f_CMCC104003	ML tree	Cui et al., 2013		2.ANT3f	Inner Mongolia, China
2.ANT3g_CMCC51020	ML tree	Cui et al., 2013		2.ANT3g	Jilin, China
2.ANT3h_CMCC106002	ML tree	Cui et al., 2013		2.ANT3h	Inner Mongolia, China
2.ANT3i_CMCC64001	ML tree	Cui et al., 2013		2.ANT3i	Inner Mongolia, China
2.ANT3j_H1959004	ML tree	Cui et al., 2013		2.ANT3j	Jilin, China
2.ANT3k_5761	ML tree	Cui et al., 2013		2.ANT3k	St.Petersbg, Russian Federation
2.ANT3l_735	ML tree	Cui et al., 2013		2.ANT3l	St.Petersbg, Russian Federation
2.MED0_C-627_KM919	ML tree	Kutyrev et al., 2018		2.MED0	Russian Federation
2.MED1_1045	ML tree	Zhgenti et al., 2015		2.MED1	Azerbaijan
2.MED1_1116-D	ML tree	Kutyrev et al., 2018		2.MED1	Russian Federation
2.MED1_1240	ML tree	Kutyrev et al., 2018		2.MED1	Azerbaijan
2.MED1_139	ML tree	Kutyrev et al., 2018		2.MED1	Taukum desert,Kazakhstan
2.MED1_173	ML tree	Kutyrev et al., 2018		2.MED1	Mangyshlaksky desert, Kazakhstan
2.MED1_1906	ML tree	Kutyrev et al., 2018		2.MED1	Precaspian sandy, Russian Federation

Supplementary Table 2 (continued)

2.MED1_244	ML tree	Kutyrev et al., 2018		2.MED1	North-Aral desert, Kazakhstan
2.MED1_261	ML tree	Kutyrev et al., 2018		2.MED1	Kobystan plain-piedmont, Azerbaijan
2.MED1_2944	ML tree	Zhgenti et al., 2015		2.MED1	Russian Federation
2.MED1_44	ML tree	Kutyrev et al., 2018		2.MED1	Azerbaijan
2.MED1_A-1809	ML tree	Eroshenko et al. 2017		2.MED1	Talas high-mountain, Kyrgyzstan
2.MED1_A-1825	ML tree	Kutyrev et al., 2018		2.MED1	Kazakhstan, Turkmenistan
2.MED1_A-1920	ML tree	Kutyrev et al., 2018		2.MED1	Pribalkhashsky desert, Kazakhstan
2.MED1_C-791	ML tree	Kutyrev et al., 2018		2.MED1	Russian Federation
2.MED1_KM816	ML tree	Kutyrev et al., 2018		2.MED1	Karakum desert, Turkmenistan
2.MED1_KM918	ML tree	Kutyrev et al., 2018		2.MED1	Russian Federation
2.MED1_M-1448	ML tree	Kutyrev et al., 2018		2.MED1	Trans-Ural steppe, Kazakhstan
2.MED1_M-1453	ML tree	Kutyrev et al., 2018		2.MED1	Ural-Embensky desert, Kazakhstan
2.MED1_M-1484	ML tree	Kutyrev et al., 2018		2.MED1	Kazakhstan
2.MED1_M-1524	ML tree	Kutyrev et al., 2018		2.MED1	Mujunkumsky desert, Kazakhstan
2.MED1_M-1763	ML tree	Kutyrev et al., 2018		2.MED1	Aral-Karakum desert, Kazakhstan
2.MED1_M-1773	ML tree	Kutyrev et al., 2018		2.MED1	Kazakhstan
2.MED1_M-1864	ML tree	Kutyrev et al., 2018		2.MED1	Precaspian sandy, Russian Federation
2.MED1_M-519	ML tree	Kutyrev et al., 2018		2.MED1	Kopetdagsky desert, Turkmenistan
2.MED1_M-549	ML tree	Kutyrev et al., 2018		2.MED1	Uzbekistan, Turkmenistan
2.MED1_M-595	ML tree	Kutyrev et al., 2018		2.MED1	Precaspian sandy, Russian Federation
2.MED1_M-978	ML tree	Kutyrev et al., 2018		2.MED1	Mangyshlaksky desert, Kazakhstan
2.MED1a_KIM	ML tree	NC_004088		2.MED1a	Iran/Kurdistan
2.MED1b_2506	ML tree	Cui et al., 2013		2.MED1b	Xinjiang, China
2.MED1c_2654	ML tree	Cui et al., 2013		2.MED1c	Xinjiang, China
2.MED1d_2504	ML tree	Cui et al., 2013		2.MED1d	Xinjiang, China
2.MED2a_I160001	ML tree	Cui et al., 2013		2.MED2a	Xinjiang, China
2.MED2b_91	ML tree	Cui et al., 2013		2.MED2b	Xinjiang, China
2.MED2c_K11973002	ML tree	Cui et al., 2013		2.MED2c	Xinjiang, China
2.MED2d_A1973001	ML tree	Cui et al., 2013		2.MED2d	Xinjiang, China
2.MED2e_7338	ML tree	Cui et al., 2013		2.MED2e	Xinjiang, China

Supplementary Table 2 (continued)

2.MED3a_J1963002	ML tree	Cui et al., 2013		2.MED3a	Gansu, China
2.MED3b_CMCC125002	ML tree	Cui et al., 2013		2.MED3b	Ningxia, China
2.MED3c_I1969003	ML tree	Cui et al., 2013		2.MED3c	Ningxia, China
2.MED3d_J1978002	ML tree	Cui et al., 2013		2.MED3d	Ningxia, China
2.MED3e_H1958004	ML tree	Cui et al., 2013		2.MED3e	Jilin, China
2.MED3f_I1970005	ML tree	Cui et al., 2013		2.MED3f	Inner Mongolia, China
2.MED3g_CMCC99103	ML tree	Cui et al., 2013		2.MED3g	Inner Mongolia, China
2.MED3h_CMCC90027	ML tree	Cui et al., 2013		2.MED3h	Inner Mongolia, China
2.MED3i_CMCC92004	ML tree	Cui et al., 2013		2.MED3i	Inner Mongolia, China
2.MED3j_I2001001	ML tree	Cui et al., 2013		2.MED3j	Shaanxi, China
2.MED3k_CMCC12003	ML tree	Cui et al., 2013		2.MED3k	Qinghai, China
2.MED3l_I1994006	ML tree	Cui et al., 2013		2.MED3l	Hebei, China
2.MED3m_SHAN11	ML tree	Cui et al., 2013		2.MED3m	Shaanxi, China
2.MED3n_SHAN12	ML tree	Cui et al., 2013		2.MED3n	Shaanxi, China
2.MED3o_I1991001	ML tree	Cui et al., 2013		2.MED3o	Inner Mongolia, China
2.MED3p_CMCC107004	ML tree	Cui et al., 2013		2.MED3p	Inner Mongolia, China
3.ANT1a_7	ML tree	Cui et al., 2013		3.ANT1a	Qinghai, China
3.ANT1b_CMCC71001	ML tree	Cui et al., 2013		3.ANT1b	Gansu, China
3.ANT1c_C1976001	ML tree	Cui et al., 2013		3.ANT1c	Gansu, China
3.ANT1d_71021	ML tree	Cui et al., 2013		3.ANT1d	Gansu, China
3.ANT2a_MGJZ6	ML tree	Cui et al., 2013		3.ANT2a	Dornogovi, Mongolia
3.ANT2b_MGJZ7	ML tree	Cui et al., 2013		3.ANT2b	Dornogovi, Mongolia
3.ANT2c_MGJZ9	ML tree	Cui et al., 2013		3.ANT2c	Govi-Altai, Mongolia
3.ANT2d_MGJZ11	ML tree	Cui et al., 2013		3.ANT2d	Bayan-Ölgii, Mongolia
3.ANT2e_MGJZ3	ML tree	Cui et al., 2013		3.ANT2e	Govi-Altai, Mongolia
4.ANT_1454	ML tree	Kutyrev et al., 2018		4.ANT	Russian Federation
4.ANT_338	ML tree	Kutyrev et al., 2018		4.ANT	Russian Federation
4.ANT_517	ML tree	Kutyrev et al., 2018		4.ANT	Russian Federation
4.ANT_KM932_I-3223	ML tree	Kutyrev et al., 2018		4.ANT	Tuva mountain, Russian Federation
4.ANT_M-1944	ML tree	Kutyrev et al., 2018		4.ANT	Tuva mountain, Russian Federation

Supplementary Table 2 (continued)

4.ANT1a_MGJZ12	ML tree	Cui et al., 2013		4.ANT1a	Bayan-Ölgii, Mongolia
Altenerding	ML tree	Feldman et al., 2016	426-571	Ancient plague	Altenerding, Germany
DIT003.B	ML tree	Keller et al., 2019	550–700	Ancient plague	Dittenheim, Germany
EDI001.A	ML tree	Keller et al., 2019	500–650	Ancient plague	Edix Hill, Great Britain
EDI003.A	ML tree	Keller et al., 2019	500–650	Ancient plague	Edix Hill, Great Britain
EDI004.A	ML tree	Keller et al., 2019	500–650	Ancient plague	Edix Hill, Great Britain
LSD001.A	ML tree	Keller et al., 2019	530–1200	Ancient plague	Le Pressoir, France
LSD019.A	ML tree	Keller et al., 2019	530–1200	Ancient plague	Le Pressoir, France
LSD020.A	ML tree	Keller et al., 2019	530–1200	Ancient plague	Le Pressoir, France
LSD021.A	ML tree	Keller et al., 2019	530–1200	Ancient plague	Le Pressoir, France
LSD023.A	ML tree	Keller et al., 2019	530–1200	Ancient plague	Le Pressoir, France
LVC	ML tree	Keller et al., 2019	400–600	Ancient plague	Lunel-Viel, France
OSL1	ML tree	Namouchi et al., 2018	1349-1350	Ancient plague	Oslo, Norway
PET004.A	ML tree	Keller et al., 2019	530–730	Ancient plague	Petting, Germany
RISE505	ML tree	Rasmussen et al., 2015	BC 1746-1626	Ancient plague	Russian Federation
RISE509	ML tree	Rasmussen et al., 2015	BC 2887-2677	Ancient plague	Bateni Afanasievo, Russian Federation
RT5	ML tree	Spyrou et al., 2018	~3800 BP	Ancient plague	Samara region, Russian Federation
TRP002.A	ML tree	Spyrou et al., 2019	1347-1350	Ancient plague	Toulouse, France
UNT003.A	ML tree	Keller et al., 2019	525–680	Ancient plague	Unterthürheim, Germany
UNT004.A	ML tree	Keller et al., 2019	525–680	Ancient plague	Unterthürheim, Germany
VAL001.B	ML tree	Keller et al., 2019	500–700	Ancient plague	Valencia, Spain
Y_pseudotuberculosis_IP32953 (outgroup)	ML tree	NC_006155		Y. pseudotuberculosis IP32953	

Supplementary Table 3. Data on genome coverage in human plague-positive samples after target enrichment

Sample Name	Nmb of reads after C&M prior mapping	Mapped Reads after RMDup	Endogenous DNA (%)	Mean Coverage	Coverage >= 1X (%)	Coverage >= 3X (%)	Coverage >= 5X (%)	Average fragment length	GC content (%)
**CO92 chromosome (NC 003143.1)**									
Rostov1639	14069919	4221	1.628	0.0637	0.99	0.38	0.28	70.32	49.45
Rostov2033	32386617	868003	55.631	12.6812	94.48	93.53	92.13	67.99	46.15
Rostov2039	13910206	226632	3.783	4.3242	88.4	60.21	33.16	88.8	48.85
Azov38	16023607	454551	37.354	5.1775	91.74	76.49	52.65	53.01	46.58
Gdansk8	343180945	7908081	77.868	184.0869	95.99	95.87	95.81	108.33	47.21
Rat	17626308	913	0.435	0.0144	1.24	0.03	0.01	73.35	50.64
**pCD1 plasmid (NC 003131.1)**									
Rostov1639	14069919	27	0	0.0291	1.87	0.23	0.01	75.81	51.88
Rostov2033	32386617	32971	14.252	34.4148	97.94	97.12	96.45	73.38	44.47
Rostov2039	13910206	9157	0.475	12.8305	94.97	81.98	63.25	98.51	48.28
Azov38	16023607	27211	9.152	22.0559	97.17	95.02	93.01	56.99	44.89
Gdansk8	343180945	132181	10.481	224.7357	98.9	98.74	98.68	119.53	44.35
Rat	17626308	121	0.001	0.1427	4.12	2.04	1.57	82.96	50.56
**pMT1 plasmid (NC 003134.1)**									
Rostov1639	14069919	24	0	0.0193	1.58	0.11	0	77.25	49.62
Rostov2033	32386617	12727	18.693	9.7797	67.35	52.77	50.63	73.93	47.4
Rostov2039	13910206	9665	0.684	9.4204	92.21	74.67	49.03	93.78	50.12
Azov38	16023607	19643	13.346	11.0281	95.68	91.16	80.98	54.01	48.84
Gdansk8	343180945	174766	15.285	204.0773	96.36	96.36	96.35	112.35	49.74
Rat	17626308	70	0.001	0.0633	3.51	0.69	0.2	86.96	49.15
**pPCP1 plasmid (NC 003132.1)**									
Rostov1639	14069919	27	0	0.2066	13.11	1.65	0.04	73.59	53.12
Rostov2033	32386617	16207	13.611	144.6295	100	100	99.99	85.77	45
Rostov2039	13910206	4890	0.427	54.3572	98.03	85.46	73.09	106.85	50.34
Azov38	16023607	8249	8.058	54.4563	99.42	95.65	90.83	63.46	47.21
Gdansk8	343180945	18679	9.018	245.4897	100	100	100	126.32	45.12
Rat	17626308	118	0.001	1.0261	27.5	14.91	11.52	83.58	50.68

Supplementary Table 5. Mapping the rat sample against different Rattus and Mus species

Ref genome	Nmb of reads after C&M prior mapping	Mapped Reads after RMDup	Endogenous DNA (%)	Coverage >= 1X (%)	Coverage >= 3X (%)	Coverage >= 5X (%)	Average fragment length	GC content (%)
Mus musculus (NC_005089)	17032499	168	0.001	16.34	8.06	4.22	52.68	40.29
Rattus fuscipes (NC_014867)	17032499	490	0.004	42.44	24.88	15.35	55.95	37.84
Rattus leucopos (NC_014855)	17032499	473	0.004	40.61	22.51	13.08	55.48	37.85
Rattus norvegicu s (NC_001665.2)	17032499	569	0.004	44.66	28.51	19.48	55.69	37.55
Rattus rattus (NC_012374)	17032499	2419	0.016	99.78	98.96	94.35	62.07	38.13
Rattus norvegicus complete genome (GCF_000001895.5)	17032499	480782	2.823	1.02	0	0	62.07	41.68
Rattus rattus complete genome (GCF_011064425.1)	17032499	681206	3.999	1.78	0	0	63.86	41.64

Supplementary Table 6. Temporal signal analysis using BETS (Duchene et al. 2019 preprint*). Temporal signal in the dataset is assessed by performing Bayesian phylogenetic reconstruction using tipdating and with samples constrained to be contemporaneous (isochronous). Strong Bayes Factor support for the tipdated reconstruction indicates sufficinet temporal signal for the timescale estimation to be performed reliably.

	**log marginal likelihood (using path sampling) from pathLikelihood.delta**	**Bayes Factor - tipdated vs isochronous**	**log marginal likelihood (using stepping stone sampling) from pathLikelihood.delta**	**Bayes Factor - tipdated vs isochronous**
**Tipdated**	-4399.65	1.97E+96	-4399.40	2.65E+96
**isochronous**	-4621.38		-4621.42	

*Duchene S, Lemey P, Stadler T, Ho SYW, Duchene D, Dhanasekaran V, Baele G. 2019. Bayesian Evaluation of Temporal Signal in Measurably Evolving Populations. bioRxiv 810697; doi: https://doi.org/10.1101/810697
